# NOX4 regulates TGFβ‐induced proliferation and self‐renewal in glioblastoma stem cells

**DOI:** 10.1002/1878-0261.13200

**Published:** 2022-03-14

**Authors:** Pedro García‐Gómez, Irene Golán, Mahsa S. Dadras, Artur Mezheyeuski, Claudia Bellomo, Kalliopi Tzavlaki, Anita Morén, Jordi Carreras‐Puigvert, Laia Caja

**Affiliations:** ^1^ 8097 Department of Medical Biochemistry and Microbiology Science for Life Laboratory Biomedical Center Uppsala University Sweden; ^2^ 8097 Ludwig Cancer Research Science for Life Laboratory Biomedical Center Uppsala University Sweden; ^3^ 8097 Department of Immunology, Genetics and Pathology Rudbeck Laboratory Science for Life Laboratory Uppsala University Sweden; ^4^ 8097 Department of Pharmaceutical Biosciences Biomedical Center Uppsala University Sweden; ^5^ Present address: Brain Metastasis Group Molecular Oncology Programme Spanish National Cancer Research Center (CNIO) Madrid 28029 Spain; ^6^ Present address: 466371 Weill Cornell Medical College Brain and Mind Research Institute New York NY 10021‐5608 USA

**Keywords:** glioblastoma, NOX4, proliferation, ROS, stem cells, TGFβ

## Abstract

Glioblastoma (GBM) is the most aggressive and common glioma subtype, with a median survival of 15 months after diagnosis. Current treatments have limited therapeutic efficacy; thus, more effective approaches are needed. The glioblastoma tumoural mass is characterised by a small cellular subpopulation – glioblastoma stem cells (GSCs) – that has been held responsible for glioblastoma initiation, cell invasion, proliferation, relapse and resistance to chemo‐ and radiotherapy. Targeted therapies against GSCs are crucial, as is understanding the molecular mechanisms that govern the GSCs. Transforming growth factor β (TGFβ) signalling and reactive oxygen species (ROS) production are known to govern and regulate cancer stem cell biology. Among the differentially expressed genes regulated by TGFβ in a transcriptomic analysis of two different patient‐derived GSCs, we found NADPH oxidase 4 (*NOX4*) as one of the top upregulated genes. Interestingly, when patient tissues were analysed, *NOX4* expression was found to be higher in GSCs versus differentiated cells. A functional analysis of the role of NOX4 downstream of TGFβ in several patient‐derived GSCs showed that TGFβ does indeed induce *NOX4* expression and increases ROS production in a NOX4‐dependent manner. NOX4 downstream of TGFβ regulates GSC proliferation, and *NOX4* expression is necessary for TGFβ‐induced expression of stem cell markers and of the transcription factor nuclear factor erythroid 2‐related factor 2 (NRF2), which in turn controls the cell’s antioxidant and metabolic responses. Interestingly, overexpression of *NOX4* recapitulates the effects induced by TGFβ in GSCs: enhanced proliferation, stemness and NRF2 expression. In conclusion, this work functionally establishes NOX4 as a key mediator of GSC biology.

## Graphical abstract

In this study, we identified NOX4 as a key mediator of glioblastoma stem cells (GSC) biology. We identified NOX4 as a key player downstream of TGFβ in GSCs. NOX4‐derived reactive oxygen species regulated proliferation and stemness not only by signalling downstream of TGFβ, but also through other mechanisms independent of TGFβ. Moreover, the expression of the NOX4 protein was higher in GSC and transition cells in comparison with differentiated cells.

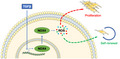

**Abbreviations**
µm: micrometre
ANOVA: analysis of variance
ARE: antioxidant response element
bFGF: basic fibroblast growth factor
BHA: butyl‐hydroxyanisole
BSA: bovine serum albumin
CL: classical
DE: differential expression
EDTA: ethylenediaminetetraacetic acid
EdU: 5‐ethynyl‐2′‐deoxyuridine
EGF: epidermal growth factor
ELDA: extreme limiting dilution analysis
FACS: fluorescence‐activated cell sorter
FDR: false discovery rate
G418: geneticin
GBM: glioblastoma
GCLM: glutamate‐cysteine ligase modifier subunit
GFAP: glial fibrillary acidic protein
GLUT1: glucose transporter type 1
GSC: glioblastoma stem cell
H_2_DCF‐DA: 2′,7′‐dichlorodihydrofluorescein diacetate
H_2_O_2_
: hydrogen peroxide
HBSS: Hanks’ balanced salt solution
HO‐1: haemoxygenase 1
IgG: immunoglobulin G
LIF: leukaemia inhibitory factor
MEM: minimum essential medium
MS: mesenchymal
NAC: *N*‐acetyl cysteine
NBDG: 2‐(*N*‐(7‐nitrobenz‐2‐oxa‐1,3‐diazol‐4‐yl)amino)‐2‐deoxyglucose
NF‐κB: nuclear factor of κ light polypeptide gene enhancer in B‐cells
NOX: NADPH oxidases
NOX4: NADPH oxidase 4
NRF2: nuclear factor erythroid 2‐related factor 2
O_2_^−^
: oxygen superoxide
OXPHOS: oxidative phosphorylation
PDGFB: platelet‐derived growth factor‐B
PFA: paraformaldehyde
PN: proneural
qPCR: quantitative polymerase chain reaction
REMBRANDT: Repository for Molecular Brain Neoplasia Data
ROS: reactive oxygen species
RT: room temperature
s.e.m: standard error of mean
siC: small interfering control
siRNA: small interfering RNA
SVZ: subventricular zone
TAC: Transcriptome Analysis Console
TGFβ: transforming growth factor
TMA: tissue microarray

## 1 Introduction

Glioblastoma (GBM) is the most prevalent and aggressive primary brain tumour. Despite current therapeutic strategies including gross surgical resection followed by treatment with radiotherapy and temozolomide [1], median survival of GBM patients is 15 months. GBM tumours are characterised by high invasiveness, vascular endothelial proliferation and a strong hypoxic component, which makes GBM refractory to radio‐ and chemotherapy [2]. Diversity is apparent at the level of malignant tissue organisation, genomic aberrations and transcript expression, resulting in three different GBM tumour groups – classical, pro‐neural and mesenchymal [3, 4, 5, 6]. Despite major advances in the study and understanding of GBM, the prognosis and treatment are still poor, which is thought to happen because of the resistance and importance of the glioblastoma stem cells (GSCs) [7]. GSCs are characterised by their self‐renewal capacity, high oncogenic potential and chemoresistance [8, 9, 10]. It is widely accepted that GBM originates from the stem cell reservoirs responsible for adult neurogenesis in the brain [2, 7, 11, 12]. Several reports support that neural stem cells localised at the subventricular zone (SVZ) are the origin of glioblastoma [13, 14]. Neural stem cell fate is controlled by environmental cues, among which cytokines play a crucial role [15]. Interestingly, the Wnt, Notch, Sonic hedgehog and transforming growth factor β (TGFβ) family signalling pathways control both GSCs and neural stem cells; being all of them involved in brain formation and neurogenesis [15, 16].

In GBM, elevated TGFβ signalling activity confers poor prognosis of the patient [17, 18]. TGFβ promotes cell proliferation in GBM, inducing the expression of platelet‐derived growth factor‐B (PDGFB) [18, 19], nodal [20] and the activation of (nuclear factor of κ light polypeptide gene enhancer in B‐cells) NF‐κB through miR‐182 [21]. Moreover, TGFβ, via its signalling mediators Smad2/3, induces the expression of the cytokine leukaemia inhibitory factor (LIF) and Sox4, which in turn enhances the expression of the stem cell transcription factor Sox2, increasing the self‐renewal capacity of the GSCs, and enhances the GSC tumour‐initiating potential, as proven *in vitro* and *in vivo* [22, 23].

Oxidative stress and malignancy progression have been correlated in several studies [24]. Reactive oxygen species (ROS) are composed mainly by hydrogen peroxide (H_2_O_2_) and oxygen superoxide (O_2_^−^) and their overproduction, which is generated by the reduction of oxygen to hydrogen peroxide, determines oxidative stress. One of the main ROS producers are the NADPH oxidases (NOX), which consist of seven different enzymes. In particular, NOX4 is constitutively active and its activity is regulated at the level of RNA and protein expression [25]. NOX‐derived ROS have several functions: they modulate transcription factors, inactivate phosphatases and help cells to migrate via the regulation of the urokinase‐type plasminogen activator expression and the regulation of matrix metalloproteases [26, 27]. NOX4 function in GBM has been related to cell growth, survival, invasion and therapeutic resistance by hypoxia‐induced radiation [28, 29].

In this work, we describe that TGFβ induces the expression of NOX4, which leads to ROS production. This is functionally important because NOX4 has a role in modulating the self‐renewal capabilities of the GSCs and their proliferation.

## 2 Materials and methods

### 2.1 Cell culture

The human GBM cell line U2987MG was previously described [30]; HepG2 and HEK293T cells were obtained from ATCC. The three cell lines were cultured in Dulbecco’s modified Eagle’s medium or Minimum Essential Medium (MEM) supplemented with 10% FBS, 2 mm l‐glutamine, 100 U·mL^−1^ penicillin and 100 μg·mL^−1^ streptomycin. The patient‐derived glioblastoma cells U3005MG‐PN, U3013MG‐PN, U3017MG‐CL, U3024MG‐MS, U3028MG‐CL, U3031MG‐MS and U3034MG‐MS were acquired from the Human Glioblastoma cell Culture resource (www.hgcc.se) at the Dept. of Immunology, Genetics and Pathology, Uppsala University, Uppsala, Sweden [31]. The cells were cultured at 37 °C (5% CO_2_ and 100% humidity) under serum‐free conditions using the N2B27 medium. In the nomenclature of the cell lines, PN stands for proneural, CL stands for classical, whereas MS stands for mesenchymal GBM subtypes. The N2B27 medium was composed of DMEM/F12 (50% v/v), Neurobasal^®^ (50% v/v), B27 without vitamin A (2% v/v), N2 (1% v/v) (Thermo Fisher Scientific, Waltham, MA, USA), Glutamine (1% v/v), Penicillin–Streptomycin (1% v/v) (Sigma‐Aldrich Co., St. Louis, MO, USA); the N2B27 medium was supplemented with EGF (10 ng·mL^−1^) and bFGF (10 ng·mL^−1^) (Peprotech, Rocky Hill, NJ, USA). U2987 GBM cells were serum starved for 6 h before TGFβ treatment. In the case of the patient‐derived cells, their N2B27 media was changed prior to TGFβ stimulation.

### 2.2 Cell transfection

For siRNA‐driven knockdowns, cells of 80% confluency were transfected with 20 nm of ON‐TARGETplus SMARTpool human NOX4 siRNA, with a pool of four different siRNAs that target several NOX4 transcript variants (L‐010194‐00‐0005), with individual sequences targeting NOX4 transcripts (J‐010194‐07‐0002, which targets 7 out 15 variants; J‐010194‐08‐0002, which targets 13 out of 15 transcript variants) and with control siRNA (D‐001810‐10‐05) (GE Healthcare Dharmacon, Lafayette, CO, USA). The transfections were done using the cationic lipid reagent silentFect™ (Bio‐Rad, Hercules, CA, USA), according to manufacturer’s instructions.

Different overexpressing stable clones were created using the U3034MG‐MS cell line with pcDNA empty plasmid and pcDNA‐V5‐NOX4, which was kindly supplied by Professor Ulla Knaus (University College of Dublin, Ireland). Using TransIT‐X2^®^ Dynamic Delivery System (Mirus, Madison, WI, USA), according to manufacturer’s instructions, 80% confluent cells were transfected and 48 h after transfection, 1 mg·mL^−1^ of Geneticin (G418) (Thermo Fisher Scientific) was added to select the positively transfected cells.

### 2.3 Intracellular reactive oxygen species measurements

Intracellular ROS measurements were done after cells were treated, then cells were incubated with 2′,7′‐dichlorodihydrofluorescein diacetate (H_2_DCF‐DA) (2.5 µm in HBSS) (Thermo Fisher Scientific) for 30 min at 37 °C. Subsequently, the cells were lysed with 250 µL of a lysis buffer composed of HEPES (25 mm and pH 7.5), MgCl_2_ (1.5 mm), NaCl (60 mm), EDTA (0.2 mm) and Triton X‐100 (1% v/v) (Sigma‐Aldrich Co.) while shaking at 4 °C during 20 min. Fluorescence was measured (485ex/520em) in an EnSpire^®^ Multimode Reader (Perkin Elmer, Waltham, MA, USA). Fluorescence values were normalised relative to the protein content and are presented as percentage of control.

### 2.4 RNA extraction, quantitative reverse transcription polymerase chain reaction (RT‐qPCR) and HTA2 affymetrix platform array

Total cellular RNA was extracted from the cells by using NucleoSpin^®^ RNA Plus kit (Macherey‐Nagel GmbH & Co. KG, Düren, Germany) according to manufacturer’s instructions. Equal amounts of total RNA were reverse‐transcribed using iScript™ cDNA Synthesis Kit (Bio‐Rad, Hercules, CA, USA) according to manufacturer’s instructions. Quantitative PCR was performed using the created cDNA in triplicates by using the CFX Connect™ Real‐Time System and CFX Manager (Bio‐Rad, Hercules, CA, USA) and KAPA SYBR^®^ FAST qPCR Kit (Kapa Biosystems, Wilmington, MA, US) according to manufacturer’s instructions. *GAPDH* was used as a reference gene. The following DNA primers (Table 1) were used.

**Table 1:** Forward and reverse primer sequences (5′–3′) used for qPCR.

Gene	Forward primer sequence	Reverse primer sequence
*NOX4*	GCAGGAGAACCAGGAGATTG	CACTGAGAAGTTGAGGGCATT
*NOX1*	CACAAGAAAAATCCTTGGGTCAA	GACAGCAGATTGCGACACACA
*LIF*	TGAACCAGATCAGGAGCCAACT	CCCCCTGGGCTGTGTAATAG
*PROM1*	ACCCAACATCATCCCTGTTCTT	ACCCAACATCATCCCTGTTCTT
*OLIG2*	CCCTCTATGGCTGTTTCTTTCTCT	TGTTGATCTTGAGACGCAGC
*NESTIN*	AGCCCTGACCACTCCAGTTTAG	CCCTCTATGGCTGTTTCTTTCTCT
*SOX2*	TGCGAGCGCTGCACAT	TCATGAGCGTCTTGGTTTTCC
*CD133*	ACCCAACATCATCCCTGTTCTT	AGCTCTTCAAGGTGCTGTTCATG
*GLUT1*	AACTCTTCAGCCAGGGTCCAC	CACAGTGAAGATGATGAAGAC
*GLUT3*	ACTTGCTGCTGAGAAGGACAT	GGGTGACCTTCTGTGTCCCC
*NRF2*	CAAAAGGAGCAAGAGAAAGCC	TCTGATTTGGGAATGTGGGC
*GCLM*	ACTAGAAGTGCAGTTGACATGG	AGGCTGTAAATGCTCCAAGG
*GAPDH*	GGAGTCAACGGATTTGGTCGTA	GGCAACAATATCCACTTTACCA

Transcriptomic analysis was performed by an HTA2 Affymetrix Platform array (Thermo Fisher Scientific). For each condition, triplicates were analysed by the Swegene centre for Integrative Biology at Lund University (SCIBLU). Transcriptome Analysis Console (TAC) 4.0.2 was used to perform differential gene expression analysis. Adjusted *P*‐values (*P*‐adj) for multiple testing, using Benjamini–Hochberg to estimate the false discovery rate (FDR), were calculated for final estimation of differential expression (DE) significance, genes with FDR < 0.1 and FC > 2 or < −2 were selected to perform Gene Ontology Biological Process analysis and visualise the results via R package clusterProfiler [32]. The expression profiles have been deposited to Array Express with accession number E‐MTAB‐9076.

### 2.5 Luciferase assay

HepG2 or 293T cells were transfected with luciferase‐encoding together with pCMV‐β‐galactosidase plasmids (100 ng), the latter as reference, using calcium phosphate for 48 h. These plasmids were synthetic NRF2‐binding promoter ARE‐luc, *hHO1* promoter‐luciferase, pEF‐NRF2 (these plasmids were a kind gift from K. Itoh, Hiroshaki University, Japan), and the plasmid pCDNA3‐V5‐NOX4 from U. Knaus (University College of Dublin, Ireland). Cells were stimulated with TGFβ (5 ng·mL^−1^) for 24 h. Two independent biological experiments were performed in three technical replicates per condition.

### 2.6 Immunoblot

For protein extraction, cells were scraped with PBS and centrifuged at 600 ***g*** during 5 min. The pellet was resuspended in lysis buffer on ice for 20 min. Afterwards, a centrifugation at 4 °C at 15 800 ***g*** during 15 min was done, and the supernatant was collected. The lysis buffer was composed of 0.5% v/v Triton X‐100, 0.5% m/v sodium deoxycolate, 10 mm EDTA, 20 mm tris(hydroxymethyl)aminomethane at pH 7.4 and 150 mm NaCl (Sigma‐Aldrich Co.) in ddH_2_O. Proteases inhibitors and phosphatase inhibitors were added, cOmplete™, EDTA‐free Protease Inhibitor Cocktail (Roche, Basel, Switzerland) and PhosSTOP™ (Roche). Protein concentration was measured by BCA. Proteins were analysed by sodium dodecyl sulphate–polyacrylamide gel electrophoresis and detected by immunoblotting. Acrylamide gels used in this study were Stain‐ Free precast gels (Bio‐Rad, Sundbyberg, Sweden), as well as homemade gels. The primary antibodies against the following proteins were used: NOX4, kindly supplied by Isabel Fabregat [33] (1 : 1000, rabbit); GLUT1 (1 : 500, rabbit, nb110‐39113; Novus Biologicals, Abingdon, UK); LIF (1 : 1000, rabbit, GTX101021; GeneTex, Alton Pkwy Irvine, CA, USA); NESTIN (1 : 2000, rabbit. ab105389; Abcam^®^, Cambridge, UK); NOTCH1 (1 : 1000, rabbit, sc‐6014; Santa Cruz Biotechnology, Heidelberg, Germany); NRF2 (1 : 1000, rabbit, GTX103322; GeneTex); and SOX2 (1 : 1000, rabbit, AB5603; Millipore, Solna, Sweden). For loading controls, the following antibodies were used GAPDH (1 : 10000, AM4300; Thermo Fisher Scientific) or β‐ACTIN (1 : 1000, mouse, sc‐69879; Santa Cruz Biotechnology, Heidelberg, Germany). Immunoblot quantification was done versus total protein normalisation when Stain‐Free precast gels were used, or versus GAPDH or β‐actin when we used homemade gels.

### 2.7 Sphere formation

Briefly, cells were seeded in Corning^®^ Costar^®^ Ultra‐Low attachment 96‐well plate (Corning Incorporated, Corning, NY, USA) performing a serial dilution from 64 to 1 cell for U3017MG, U3031MG and U3034MG cells, performed in 12 or 7 replicates in 100 µL, respectively. Cells were incubated for 6 days after NOX4 silencing with or without TGFβ1 treatment (5 ng·mL^−1^); then, the wells with neurospheres > 50 µm were counted as positive. The neurospheres were visualised using a phase‐contrast Axiovert 40 CFL microscope (Carl‐Zeiss, Oberkochen, Germany). The data were processed by the R package Extreme Limiting Dilution Analysis (ELDA) [34].

The sphere capacity formation was also assessed by counting the number of spheres formed by U3017MG and U3031MG after silencing of NOX4 with or without TGFβ; or by U3034MG overexpressing clones. Briefly, 1000 cells in 1 mL were seeded in Corning^®^ Costar^®^ Ultra‐Low attachment 24‐well plate (Corning Incorporated, Corning, NY, USA). Cells were incubated for 6 days; then, the neurospheres > 50 µm were counted.

### 2.8 Proliferation assays

#### 2.8.1 Ki67 staining

Cells were treated with DMSO (1% v/v), TGFβ1 (5 ng·mL^−1^), and/or NOX1/4 inhibitor (GKT137831) (20 µm) during 24h in N2B27 medium. After treatment, cells were washed with PBS and fixed with 3.7% (w/v) paraformaldehyde in PBS for 15 min. Subsequently, cells were washed with PBS and blocked with 10% FBS in PBS with 1% BSA for 1 h, at RT. Then, cells were permeabilised with 0.1% Triton‐X‐100 in 0.1% BSA for 5 min. Next, cells were incubated at RT for 2 h with primary antibodies against Ki67 (1 : 1000 in PBS containing 1% BSA, rabbit, ab15580; Abcam^®^). Afterwards, cells were washed in PBS and incubated for 1 h in dark at RT with donkey anti‐Rabbit IgG Alexa Fluor 488 secondary antibody (1 : 200 in PBS with 1% BSA, A21206; Thermo Fisher Scientific). Then, cells were incubated in dark at RT for 5 min with DAPI (1 : 1000 in PBS with 1% BSA; Sigma‐Aldrich Co.). Later, cells were washed three times with PBS and the coverslips were mounted on the slides using Fluoromont‐G^®^ mounting medium (Southern Biotech, Birmingham, AL, USA) and dried in dark at RT. Pictures were acquired with the fluorescence microscope Eclipse 90i and processed using NIS‐Elements software (Nikon, Tokyo, Japan). The quantification of the proliferative phenotype of cells was performed by quantifying Ki67‐positive cells, and total number of nuclei per picture using fiji image j software [35].

#### 2.8.2 EdU click chemistry

5‐ethynyl‐2′‐deoxyuridine (EdU) staining was conducted using a Click‐iT imaging kit (Thermo Fisher Scientific) according to the manufacturer’s protocols. Briefly, 10 000 cells/well were seeded in a 96‐black well plate. When using U2987 cells, the next day cells were starved for 6 h and treated with TGFβ1 (5 ng·mL^−1^), and/or NOX1/4 inhibitor (GKT137831) (20 µm). In the case of U3024MG or U3031MG cells, N2B27 media was changed before treating the cells with TGFβ1 (5 ng·mL^−1^), and/or NOX1/4 inhibitor (GKT137831) (20 µm). Cells were treated for 24 h and incubated with Edu the last 6 h of treatment, then cells were fixed with 4% PFA for 15 min and subsequently permeabilised with 0.5% Triton X‐100 for 10 min at room temperature. After washing with PBS, the cells were incubated with a Click‐iT reaction cocktail (Click‐iT reaction buffer, CuSO4, reaction buffer additive and Alexa Fluor 647 azide) for 30 min. After staining with Hoechst 33342, images were acquired for quantitative analysis with an ImageXpress XLS (Molecular Devices) high‐content microscope. EdU positive cells were identified using cellprofiler [36]; briefly, each nucleus was detected using Hoechst staining signal, which allowed then to quantify Edu signal within the nuclei and identify the percentage of EdU positive cells.

#### 2.8.3 MTS assay

Proliferation of 1000 cells/well seeded in a 96‐well plate was assessed using MTS assay. Next day after seeding, U2987 cells were starved for 6 h and treated with TGFβ1 (5 ng·mL^−1^) and/or NOX1/4 inhibitor (GKT137831) (20 µm) or BHA (200 µm). Proliferation was monitored at 1, 3 and 5 days after treatment by MTS assay, following the manufacturer’s protocol (Promega, Biotech AB, Nacka, Sweden). In the case of U3034MG cells, cells were seed and cultured with N2B27 media, proliferation was monitored at 1, 3 and 5 days after seeding of the cells by MTS assay.

### 2.9 Flow cytometry measurements: cell death, cell cycle, CD44 and glucose uptake measurement

U3031MG cells were transiently transfected with control (siControl) or NOX4 (siNOX4) siRNAs and stimulated with TGFβ1 for 24h. For cell death measurement, adherent cells and floating dead cells were harvested using accutase and centrifuged for 5 min, cells were then resuspended in 100 µL of 5% FBS/PBS with 2.5 µL of FITC Annexin V (BioLegend, San Diego, CA, USA) and 0.6 µL of Draq7 (BD Pharmingen, Stockholm, Sweden). After vortex, the cells were incubated for 15 min at RT and darkness. Finally, the volume was completed with 400 µL of 5% FBS/PBS.

To measure the glucose uptake, cells were harvested after treatment using accutase and single‐cell suspensions were incubated for 30 min at 37 °C with 100 mm 2‐NBDG (2‐(*N*‐(7‐Nitrobenz‐2‐oxa‐1,3‐diazol‐4‐yl)Amino)‐2‐Deoxyglucose) from Thermo Fisher Scientific.

To measure CD44 positive cells, cells were harvested with accutase, centrifugated 5 min and then resuspended in 50 µL of 5% BSA/PBS. The blocking was done for 15 min on ice and shacking. After that, the antibody FITC anti‐human CD44 (BioLegend) was added in a concentration of 1 : 400 and a final volume of 100 µL of 5% BSA/PBS was added and incubated for 30 min on ice, shacking and kept in darkness. The cells were centrifugated for 1 min 400 rcf and resuspended in a final volume of 300 µL of PBS. The isotyping was also done with FITC Mouse IgG1, κ Isotype Control (BD Pharmingen, Stockholm, Sweden) to ensure no unspecified union. In both cases, glucose uptake and CD44 positive cells measurement, all samples included SytoxBlue staining (1 µm; Thermo Fisher Scientific) to discard dead cells and analyse only the live cell population.

Glucose uptake, Annexin/Draq7 and CD44 analysis was performed by fluorescence‐activated cell sorter (FACS) using a Cytoflex S flow cytometer (Beckman Coulter, Bromma, Sweden), their quantification was done by using flowjo llc software version 10.7.2.

### 2.10 Multiplex immunohistochemical staining

**Fig. 1:**
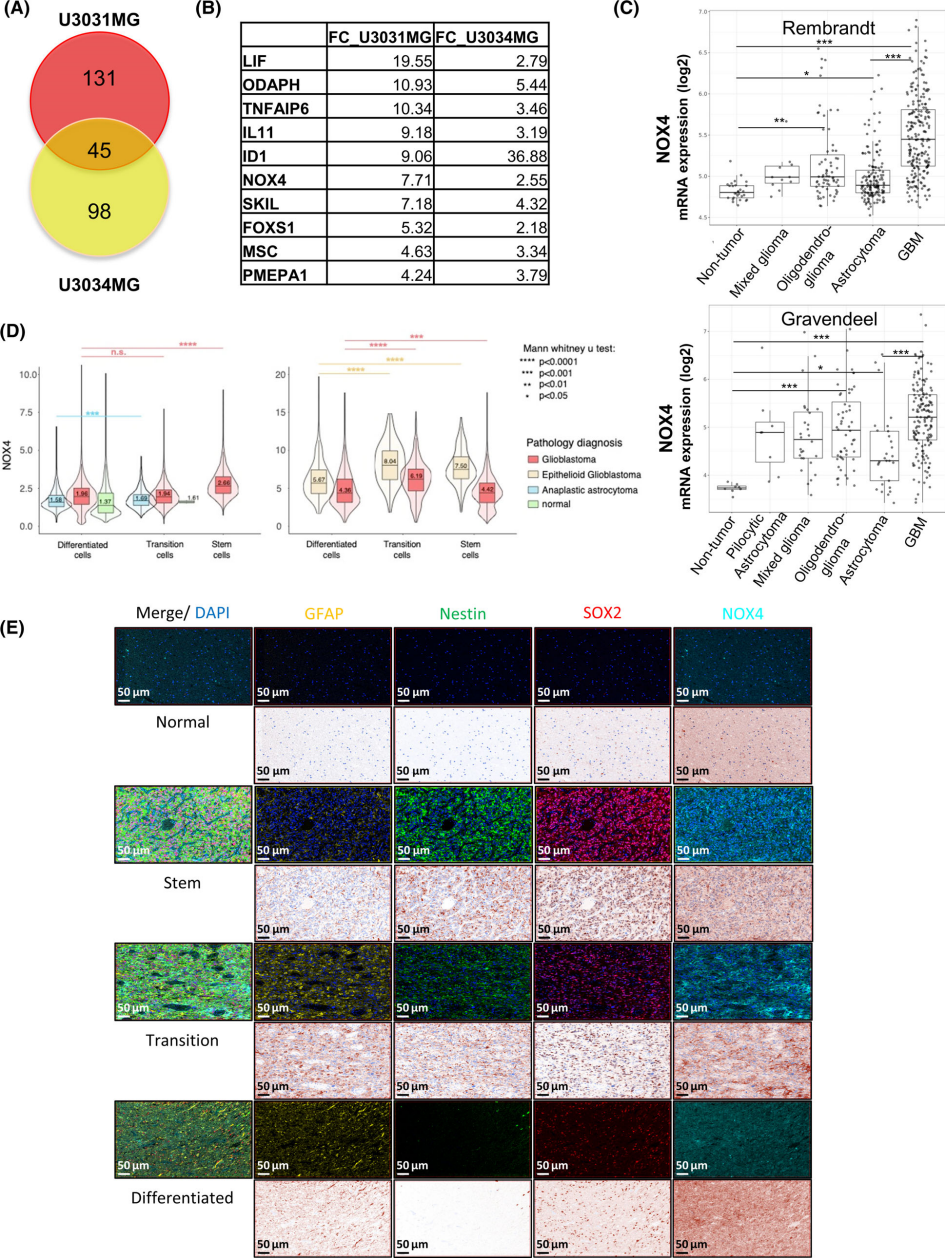
NOX4 is upregulated by TGFβ1 in Glioblastoma. (A) Venn diagram illustration of gene expression similarity between upregulated genes by TGFβ1 in U3031MG cells and U3034MG cells after 24 h of treatment. (B) Table indicating the fold‐change values of the common top 10 upregulated genes by TGFβ1 in both cell lines. (A, B) Data are extracted from the HTA2 Affymetrix Platform transcriptomic analysis performed in U3031MG and U3034MG cells treated with or without TGFβ1 in triplicate. (C) NOX4 mRNA expression in different types of glioma cells relative to non‐tumour cells using different databases (REMBRANDT, top; Gravendeel, bottom), *t*‐test with Bonferroni correction is shown in the pairwise comparisons between the indicated groups, significant differences at *P < 0.05, **P < 0.01, ***P < 0.001. (D) NOX4 expression per cell in normal, glioblastoma and anaplastic astrocytoma tissue samples from two different tissue microarrays (colour‐coded) plotted as a function of the three marker proteins expression in the same cell. Cells were classified to three groups, GBM_diff (GFAP^high^/Nestin^low^/SOX2^low^), GBM_transition (GFAP^medium^/Nestin^medium^/SOX2^medium^) and GBM_Stem (GFAP^low^/Nestin^high^/SOX2^high^). Statistical comparison indicates ***P < 0.001, ****P < 0.0001, statistics: Mann–Whitney *U* test. (E) Representative images displaying staining of the four proteins, NOX4/Nestin/SOX2/GFAP, in normal brain (four cases in duplicate) and glioblastoma tissue samples (36 cases in duplicate), scale bar indicates 50 µm.

We analysed two tissue microarray (TMA) with normal brain, glioblastoma, anaplastic glioblastoma and epithelioid glioblastoma tissue samples (GL806d, GL806f, from US Biomax, Derwood, MD, USA) by mutiplexed immunohistochemical analysis as previously described [37]. The primary antibodies used were: rabbit anti‐NOX4, kindly supplied by Isabel Fabregat (1 : 100), mouse anti‐Nestin (1 : 800, NB300‐266; Novus Biologicals Abingdon, UK), rabbit anti‐GFAP (1 : 50; sc‐6171‐R; Santa Cruz Biotechnology Inc., Santa Cruz, CA, USA) and rabbit anti‐SOX2 (1 : 100; ab5603; Merck/Sigma, Stockholm, Sweden), rabbit‐CD44 (1 : 100, ab157107; Abcam^®^). Image analysis (inform software; Akoya) and R software, version 3.3.3 [38, 39] were used to classify cells according to marker expression patterns, as described earlier [40]. In short, a number of cells were visually assessed to define minimal level of the expression of each marker to be considered as positive; the expression levels below these thresholds were considered as background and respective cells were classified as low expressing for such marker (i.e. Nestin^low^). Further, the expression range from this threshold to the maximal marker expression was split by median, making marker‐medium group and marker‐high group. Using this data, three classes of tissue cells were generated according to Nestin, SOX2 and GFAP expression in Fig. 1: GBM_diff (GFAP^high^/Nestin^low^/SOX2^low^) GBM_transition (GFAP^medium^/Nestin^medium^/SOX2^medium^) and GBM_Stem (GFAP^low^/Nestin^high^/SOX2^high^); and in Fig. S2 cells were classified as follows: GBM_diff (CD44^low^/Nestin^low^/SOX2^low^), GBM_transition (CD44^medium^/Nestin^medium^/SOX2^medium^) and GBM_Stem (CD44^high^/Nestin^high^/SOX2^high^). NOX4 expression was evaluated then in these cells as a continuous variable (without applying thresholds). In the case of SOX2, only the nuclear staining was used to evaluate this marker expression.

### 2.11 Statistics

The statistical analysis was performed by using the software graphpad prism ^®^ v.7 (GraphPad Software, San Diego, CA, USA). The data are presented as the average ± standard error of the mean (SEM). Most statistical tests used were one‐way or two‐way ANOVA followed by Dunnett’s post‐test or Bonferroni post‐test. A *P*‐value < 0.05 was considered as statistically significant. The significance was considered as *P < 0.05, **P < 0.01 and ***P < 0.001, as it is indicated in each figure.

## 3 Results

### 3.1 TGFβ induces NOX4 in glioblastoma stem cells

In order to study mechanisms by which TGFβ regulates GBM stemness, we used established GSCs derived from two different GBM patients classified as mesenchymal subtype, U3031MG and U3034MG [31]. These cells were cultured in stem cell media with or without TGFβ for 24 h, their RNA was extracted and a transcriptomic analysis using the HTA2 Affymetrix platform was performed. TGFβ induced 176 and 143 genes in U3031MG and U3034MG, respectively; while it downregulated 172 and 131 genes in U3031MG and U3034MG, respectively. In the group of TGFβ‐upregulated genes, a subset of 45 genes was common between U3031MG and U3034MG cells (Fig. 1A, Table S1). These genes functions were related to the TGFβ response, the regulation of animal organ morphogenesis and cell differentiation, including the epithelial‐to‐mesenchymal transition process (Fig. S1a). Among the top 10 upregulated genes we detected known genes modulated by TGFβ in GBM, such as *LIF* and *ID1* [23, 41], as well as *NOX4* (Fig. 1B). We focused on NOX4, an enzyme responsible to produce ROS, which has been described to contribute to apoptosis, migration, invasion and differentiation [24]. The role of NOX4 has been previously studied in the TGFβ signalling pathways in different tumours [42, 43], but its role downstream of TGFβ in GBM, and specifically in GSCs, is still unknown.

Analysis of NOX4 expression in different glioma subtypes using the Gliovis data portal for visualisation [44] revealed differences between tumour and non‐tumour cells, with highest expression of NOX4 recorded in GBM and in grade IV tumours compared with low‐grade glioma tumours (Fig. 1C, Fig. S1b) in two different datasets: the Repository for Molecular Brain Neoplasia Data (REMBRANDT) [45] and the Gravendeel dataset [46]. Correlating this expression with survival expectancy in glioma and GBM, a worse prognosis was recorded in the patients with higher levels of NOX4 compared with patients with low NOX4 expression (Fig. S1c). Moreover, a positive correlation was observed between different TGFβ family members and NOX4 expression (Fig. S1d).

To analyse the expression of NOX4 in tissues from GBM patients, we performed multiplex immunohistochemistry analysis on two tissue microarrays (TMA) with human GBM, anaplastic astrocytoma, epithelioid glioblastoma, and non‐tumoural brain samples. We performed co‐staining with multiple antibodies, aiming at linking the patient NOX4 protein expression with the GSC subpopulation and the differentiated GBM cells (Fig. 1D,E, Fig. S2). In addition to NOX4, we used antibodies against Nestin and SOX2, which are established stem‐cell markers in GBM, and GFAP as an indicator of the astrocytic lineage in TMA1 (Fig. 1D, left); in the second TMA (Fig. 1D, right, Fig. 1E, Fig. S2) we added CD44 antibody to better identify stem cells. We then classified each cell in the different tissue sections into to three groups: GBM_diff (GFAP^high^/Nestin^low^/SOX2^low^) representing GBM cells differentiated towards the astrocytic lineage, GBM_transition (GFAP^medium^/Nestin^medium^/SOX2^medium^) possibly representing tumour cells undergoing differentiation transitions, and GBM_Stem (GFAP^low^/Nestin^high^/SOX2^high^) representing the GSCs. We converted each fluorescent channel to the same brown pseudo‐colour that resembles traditional immunohistochemical staining. Immunostaining of normal brain tissue showed low levels of the stem cell markers Nestin and SOX2, GFAP was mainly also expressed at low level in normal brain cells (Fig. 1E). Interestingly, in the GBM tissue samples, the GBM cells with high GFAP (GBM‐differentiation group) exhibited the lowest NOX4. In contrast, NOX4 expression levels were higher in GBM transition cells and GBM_stem cells (Fig. 1D,E). Next, we classified the cells only according to the stem cell markers CD44, Nestin, and SOX2 into GBM_diff (CD44^low^/Nestin^low^/SOX2^low^), GBM_transition (CD44^medium^/Nestin^medium^/SOX2^medium^) and GBM_Stem (CD44^high^/Nestin^high^/SOX2^high^). Using only stem cell markers to classify the cells, we also observed that NOX4 levels were higher in transition and stem GBM cells both in glioblastoma and epithelioid glioblastoma patients, compared with the differentiated GBM cells (Fig. S2). At the RNA level, we queried whether there was also a positive association between the expression of different stem cell markers (CD44, Nestin, SOX2 or LIF) and NOX4 expression in GBM patients from the REMBRANDT and the Granvendel databases. Indeed, we observed that there is a positive correlation between CD44, Nestin and LIF with NOX4 expression; however, SOX2 had a positive correlation with NOX4 expression only in the REMBRANDT dataset (Fig. S1e). Overall, our protein data and our *in silico* analysis show that NOX4 is mainly expressed in GBM transition and stem cells, and its expression is lower in the bulk differentiated tumour cells.

### 3.2 TGFβ stimulates ROS production in a NOX4‐dependent manner

**Fig. 2:**
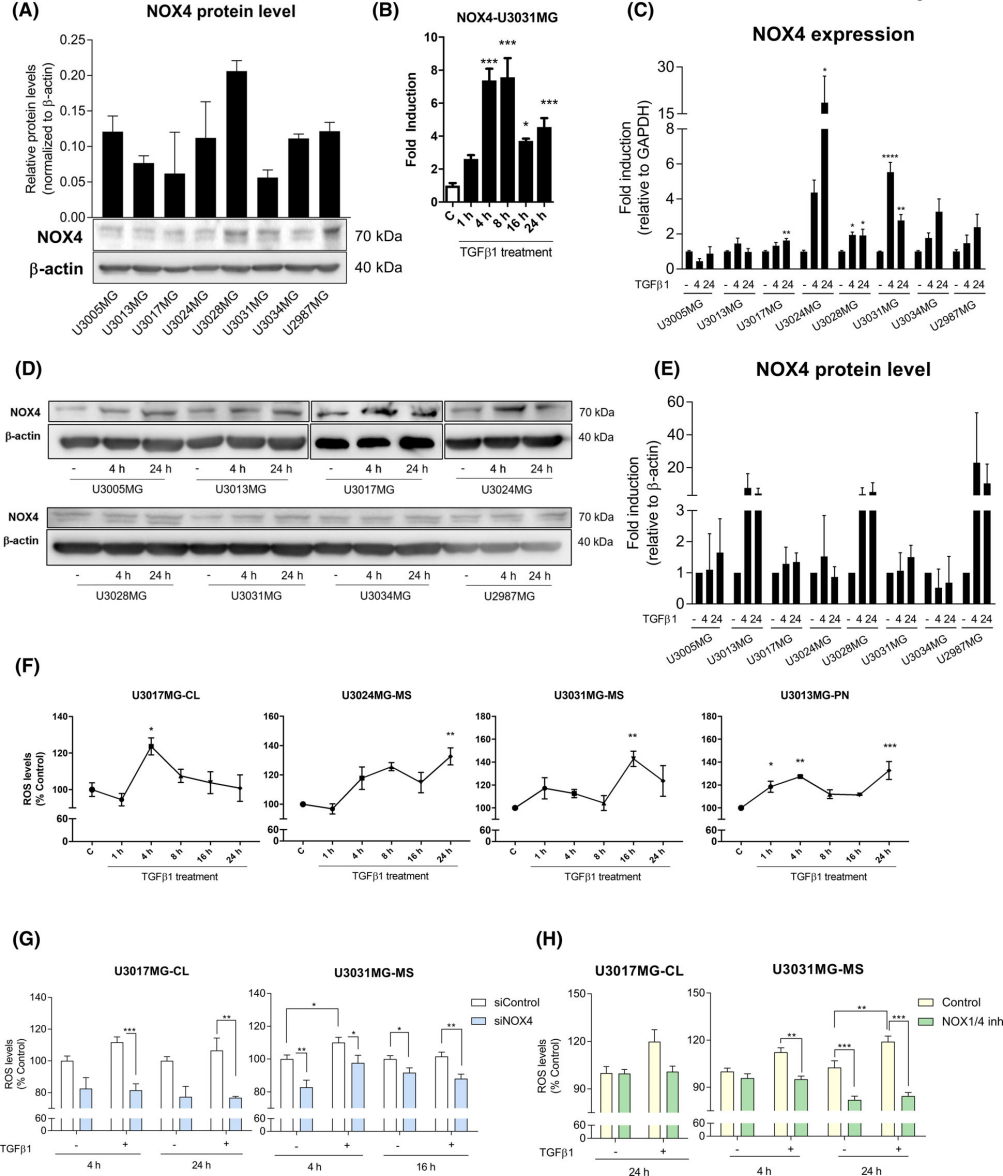
TGFβ1 increases the reactive species of oxygen in a NOX4‐dependent manner. (A) Expression panel of *NOX4* in different patient‐derived glioblastoma stem cells lines at the protein level, data represent the mean ± SEM (*n* = 2 independent experiment). (B) Time course of NOX4 mRNA expression levels analysed by qPCR in U3031MG cell line upon TGFβ1 treatment, representative experiment of 2; statistics: one‐way ANOVA test, Tukey’s multiple comparisons test. (C) *NOX4* mRNA expression levels analysed by qPCR in different cell lines and time points upon TGFβ1 stimulation. Data represent the mean ± SEM (*n* = 3 independent experiments); statistics: one‐way ANOVA test, Dunnett's multiple comparisons test; (D) representative immunoblot of NOX4 protein in different cell lines upon TGFβ1 stimulation, β‐actin was used as loading control, (E) the quantification of two experiments is plotted as mean ± SEM; statistics: one‐way ANOVA test, Dunnett's multiple comparisons test. (F) Time course of ROS production upon TGFβ1 treatment in different cell lines. Data represent the mean ± SEM (*n* = 2 independent experiments, each with biological triplicate); statistics: one‐way ANOVA test, Tukey’s multiple comparisons test. (G) ROS production upon TGFβ1 stimulation in control (siControl) and NOX4‐silenced (siNOX4, pool of four siRNA sequences) cells in different cell lines and time points. Data represent the mean ± SEM (*n* = 3 independent experiments and each with biological triplicate); statistics: two‐way ANOVA test, Bonferroni posttests. (H) ROS production upon TGFβ1 stimulation in the presence or absence of NOX1/4 inhibitor (GKT137831, 20 µm) in different GSC cells and time points. Data represent the mean ± SEM (*n* = 2 independent experiments, each with biological triplicate); statistics: two‐way ANOVA test, Sidak’s multiple comparison. In the cell line names, CL stands for classical subtype, PN stands for proneural subtype and MS for mesenchymal subtype of GBM. Statistical comparison indicates *P < 0.05, **P < 0.01, ***P < 0.001, ****P < 0.0001.

As NOX4 is highly expressed in GSCs, we analysed NOX4 expression in different patient‐derived GSC cell lines, revealing differences in NOX4 expression both at the RNA and protein level, possibly reflecting individual patient diversity (Fig. 2A, Fig. S3a). TGFβ induced the expression of NOX4 at different time points in all GSCs cell lines from distinguished subtypes both at the RNA and protein level (Fig. 2B–E). Next, we analysed the production of ROS in the GSCs after TGFβ treatment, and found that ROS production increased after TGFβ treatment at different time points in the different cell lines (Fig. 2F). Interestingly, ROS production was reduced either when silencing NOX4 with specific small interfering RNAs using a pool of four different siRNA (siNOX4) or using single siRNA against NOX4 (siNOX4#7, siNOX4#8) (Fig. 2G, Fig. S3b), or when using the enzymatic activity inhibitor of NOX4 (GKT137831) (Fig. 2H). It is worth to clarify that GKT137831 inhibits both NOX1 and NOX4; however, NOX1 should not contribute to ROS production due to its very low levels or undetectable levels of expression in the used GSCs (Fig. S3a).

### 3.3 TGFβ increases GSC proliferation in a NOX4‐dependent manner

**Fig. 3:**
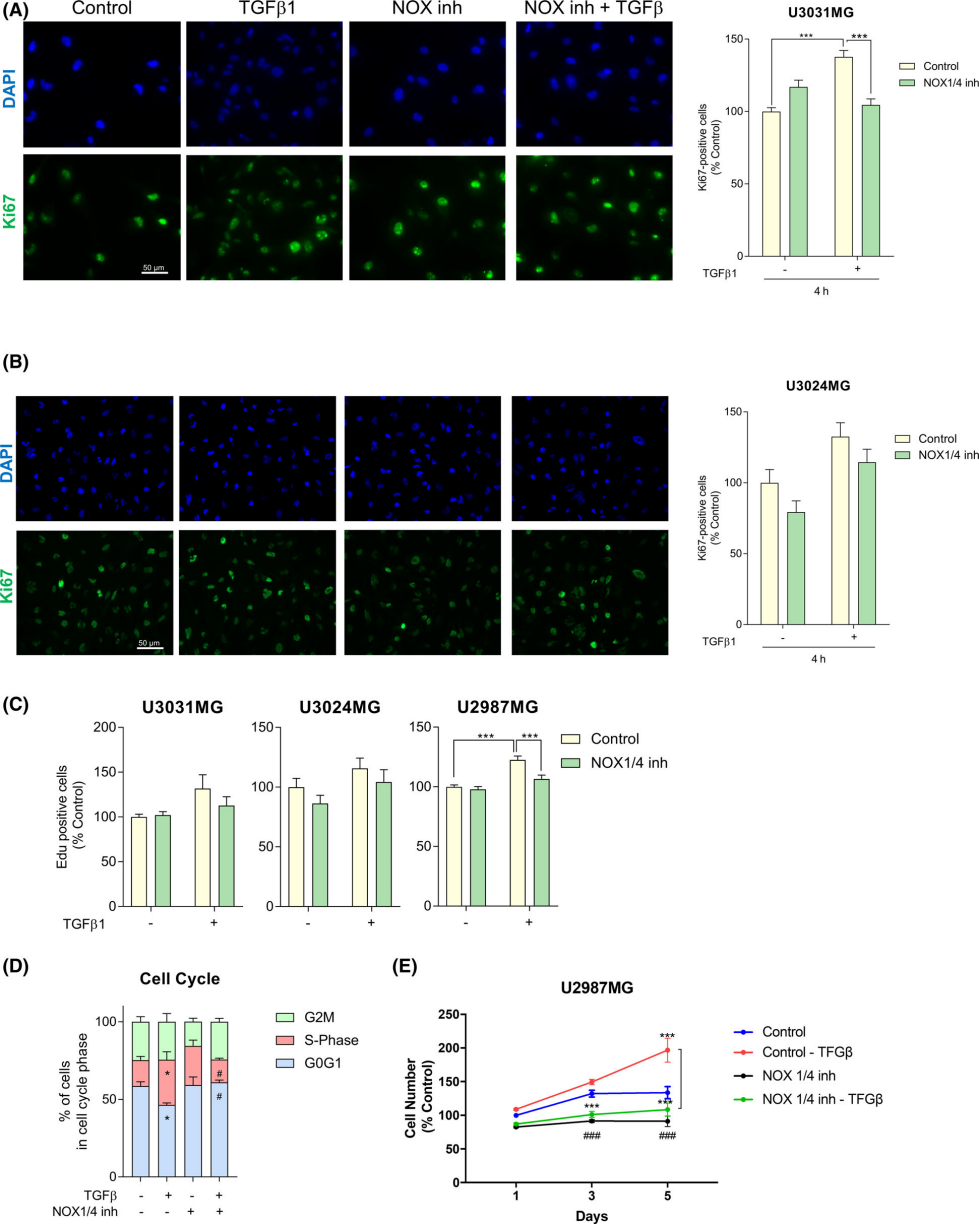
TGF‐β1 increases cell proliferation in a NOX4‐dependent manner. U3031MG (A) and U3024MG (B) cells were stimulated with TGFβ1 for 24h, in the presence or absence of the NOX1/4 inhibitor (GKT137831, 20 µm), and the following experiments were performed. (A, B) Left: Representative microscopic images of immunofluorescence of Ki67 (green) and DAPI (blue). Scale bar, 50 μm. Right: Quantification of Ki67 positive cells with respect to the control. Data represent the mean ± SEM (*n* = 2 for U3031MG and *n* = 3 for U3024MG independent experiment, 10 images per condition and experiments were quantified); statistics: two‐way ANOVA test, Sidak’s multiple comparison. (C) EdU staining was performed, data represent the mean ± SEM (*n* = 4 independent experiment with four biological replicates, nine images per condition and experiment were quantified); statistics: two‐way ANOVA test, Sidak’s multiple comparison. (D) Cell cycle was analysed by flow cytometry after staining the cells with propidium iodide, data represent the mean ± SEM (*n* = 4 independent experiment); statistics: two‐way ANOVA test, Dunnett’s multiple comparison. (E) Cell viability was assayed by MTS at the indicated times, data represent the mean ± SEM (*n* = 2 independent experiment with four biological replicates); statistics: two‐way ANOVA test, Tukey’s multiple comparison. (A–C, E) Statistical comparison indicates *P < 0.05, ***P < 0.001. (D) Statistical comparison indicates *P < 0.05, ***P < 0.001 vs Control; ^#^ *P* < 0.05, ^###^ *P* < 0.001 calculated vs Control‐TGFβ.

TGFβ is known to increase cell proliferation of different cell lineages including glioma cells [18, 47]. Moreover, ROS production generated by NOX proteins has also been positively linked to cell proliferation [48, 49]. In order to understand the role of NOX4 in cell proliferation, a Ki67 immunofluorescence assay was performed in GSCs. The percentage of Ki67‐positive nuclei, indicative of actively proliferating cells, was increased when cells were treated with TGFβ, while this event was abolished almost to basal levels when NOX4 was inhibited in two different cell lines (Fig. 3A,B). To further validate these results, we performed EdU staining in two patient‐derived GSC cell lines cultured in stem cell media (U3024MG, U3031MG), and in another GBM cell line cultured in media with 10% FBS (U2987MG), but starved in the current experiments prior to TGFβ treatment. In all cell lines, TGFβ increased the percentage of cells proliferating, and this increase was strongly or mildly decreased when cells were pre‐treated with NOX1/4 inhibitor (Fig. 3C); however, only when using the U2987MG cells we observed a significant effect by TGFβ and NOX1/4 inhibition. A robust trend was observed when using U3031MG and U3024MG cell lines towards the same phenotype, even though it was not significant. U2987MG cells are grown in media complemented with serum and starved prior to treatment, while U3031MG and U3024MG are grown in stem cell media N2B27. This difference in media composition could explain why some GBM cells respond more potently than others to TGFβ‐induced proliferation. Next, we analysed the effect of TGFβ in cell cycle and observed that in U3031MG, TGFβ significantly decreased the percentage of cells in G0/G1‐phase and increased the percentage of cells in S‐phase, effects that were lost when NOX4 was inhibited (Fig. 3D). Finally, the proliferation induced by TGFβ was sustained in time in the U2987MG cells, both basal and TGFβ‐induced proliferation were blocked when NOX4 was inhibited (Fig. 3E). Next, we analysed whether lack of NOX4 could induce cell death. When silencing NOX4, we neither observed a significant increase in the percentage of annexin‐positive cells (both early and late apoptosis, Fig. S4a) nor an increase in the percentage of late apoptosis and necrotic cells (Fig. S4b) in comparison with cells transfected with siControl, both analysed via flow cytometry. These results indicate a crucial function of NOX4 on the proliferative phenotype induced by TGFβ signalling, validating that the NOX4‐dependent generation of ROS is physiologically important.

### 3.4 TGFβ induces self‐renewal capacity of GSC in a NOX4‐dependent manner

**Fig. 4:**
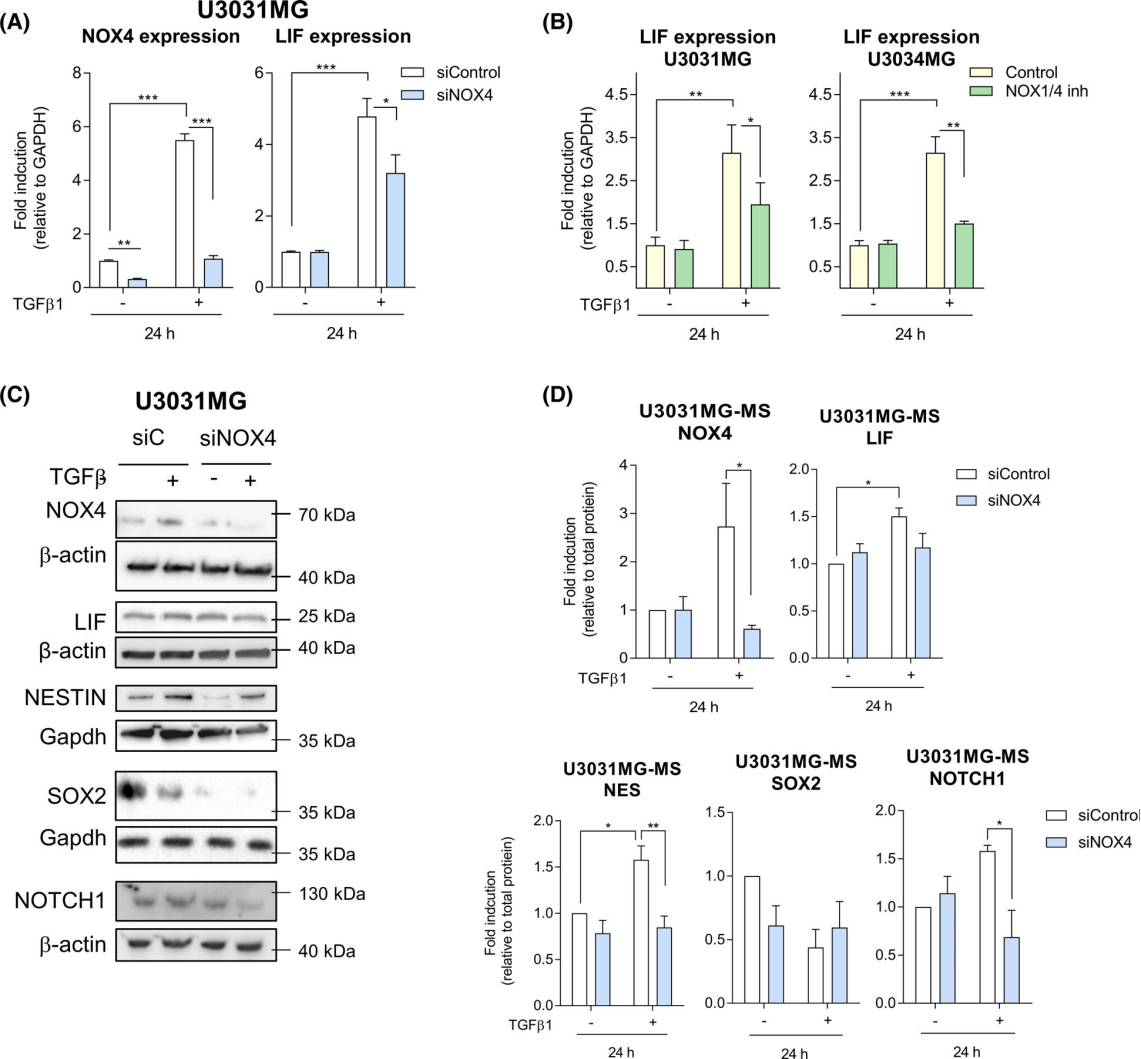
NOX4 regulates TGFβ1‐induced stem cell‐related proteins. (A) U3031MG cells were transiently transfected with control (siControl) or NOX4 (siNOX4, pool of four siRNA sequences) siRNAs and stimulated with TGFβ1 for 24 h, mRNA expression levels analysed by qPCR, data represent the mean ± SEM (*n* = 5 independent experiments and each with technical triplicate); statistics: two‐way ANOVA test, Tukey’s multiple comparison. (B) mRNA expression levels analysed by qPCR: U3031MG or U3034MG cells were stimulated with TGFβ1 for 24 h in with or without NOX1/4 inhibitor. Data represent the mean ± SEM (*n* = 2 independent experiments and each with technical triplicate); statistics: two‐way ANOVA test, Tukey’s multiple comparison. (C) U3031MG cells were transiently transfected with control (siControl) or NOX4 (siNOX4, pool of four siRNA sequences) siRNAs and stimulated with TGFβ1 for 24 h, immunoblot of the indicated proteins, Gapdh or β‐actin, are used as a loading control. (C) A representative experiment of (D) Quantification of immunoblot data using the densitometric analysis of each protein, normalised with total protein using Stain‐Free precast gels using image lab™ software (Bio‐Rad, Sundbyberg, Sweden); data represent mean ± SEM (*n* = 3–6 independent experiments, depending on the antibody), statistics: two‐way ANOVA test, Sidak’s multiple comparison. (A, B, D) Statistical comparison indicates *P < 0.05, **P < 0.01, ***P < 0.001.

**Fig. 5:**
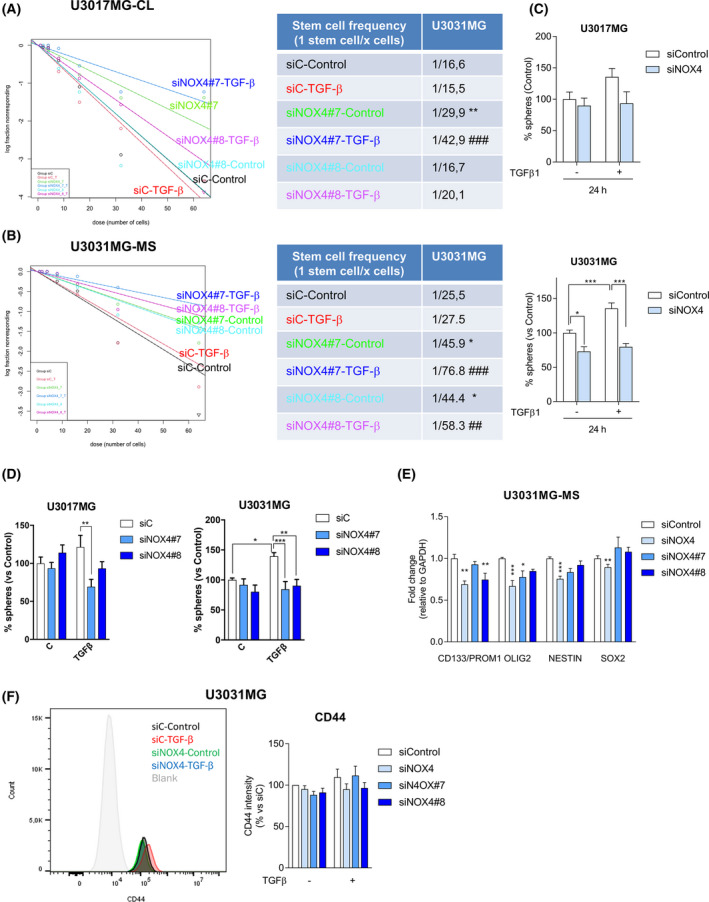
NOX4 regulates GSC self‐renewal capacity induced by TGFβ1. (A, B) U3017MG and U3031MG cells were transiently transfected with control (siControl), or two different siRNA sequences against NOX4 (siN4#7, siN4#8) and stimulated with TGFβ1, when indicated, for 6 days. Limiting dilution neurosphere assay was performed, analysed by ELDA showing stem cell frequencies. The table presents the stem cell frequency (*n* = 5 with 7–12 replicates each for a; *n* = 3 with 6 replicates in B); statistics: Chi square. (C, D) U3017MG and U3031MG cells were transiently transfected with control (siControl), NOX4 (siNOX4, pool of four siRNA sequences) siRNAs or using two different siRNA sequences against NOX4 (siN4#7, siN4#8) and stimulated with TGFβ1, when indicated, for 6 days, when the number of spheres were counted, data represent mean ± SEM (*n* = 2 for U3017MG, *n* = 6 for U3031MG in D, *n* = 3 for both cells, all experiments with four biological replicates); statistics: two‐way ANOVA test, Sidak’s multiple comparison. (E) U3031MG cells were transiently transfected with control (siControl) or NOX4 siRNAs (siNOX4, pool of four siRNA sequences) or using two different siRNA sequences against NOX4 (siN4#7, siN4#8) for 5 days; the expression levels of mRNA of the indicated genes were analysed by qPCR. Data represent the mean ± SEM (*n* = 2–4 independent experiments and each with technical triplicate); statistics: two‐Way ANOVA test, Tukey’s multiple comparison. (F). Flow cytometry analysis of CD44 surface marker expression in U3031MG transiently transfected with control (siControl), NOX4 (siNOX4, pool of four siRNA sequences) siRNAs or using two different siRNA sequences against NOX4 (siN4#7, siN4#8) and stimulated with TGFβ1 for 24 h, left shows a representative FACS profile, the graph right represents mean ± SEM (*n* = 4); statistics: two‐way ANOVA test, Tukey’s multiple comparison. (A–F) Statistical comparison indicates *P < 0.05, **P < 0.01, ***P < 0.001 vs siControl; ^##^ *P* < 0.01, ^###^ *P* < 0.001 vs siC‐TGFβ.

TGFβ not only regulates GSC proliferation but also maintains GSCs stemness and self‐renewal capacity [22, 23]. Moreover, ROS have been reported to have a role either in stem cell maintenance and differentiation, or in the self‐renewal capacity of the neural stem cells [48, 50, 51]. In order to study whether NOX4 had a role on GSC self‐renewal induced by TGFβ, we analysed the effects of NOX4 in TGFβ‐induced expression of LIF at the transcriptional level, as LIF has been previously described to be involved in TGFβ‐induced self‐renewal capacity [23]. Indeed, *LIF* was upregulated upon TGFβ treatment, but this effect was reduced when silencing or inhibiting NOX4 (Fig. 4A,B, Fig. S3d). Knockdown efficiency of NOX4 using the siRNA pool or single siRNA against NOX4 was of approximately 80% in U3031MG and of 50% in U3017MG (Fig. 4C,D, Figs S3c and S5a,b). At the protein level, we also analysed the expression of LIF, two known stem cell markers, Nestin and SOX2, as well as cleaved NOTCH1, the expression of which has been linked to glioblastoma proliferation and stemness [52, 53]. TGFβ induced the expression of Nestin, LIF and cleaved NOTCH1 but not of SOX2, while the silencing of NOX4 strongly impaired these effects and slightly decreased the basal expression of Nestin and SOX2 (Fig. 4C,D, Fig. S5a,b). The inhibition of NOX4 had similar effects, but not as strong as NOX4 silencing (Fig. S5c,d). Next, a limiting dilution neurosphere assay was performed, which revealed a high self‐renewal capacity of the patient‐derived GSCs, which made them unresponsive to TGFβ effects. Interestingly, silencing of NOX4 decreased the self‐renewal capacity in all GSCs tested (Fig. 5A,B). In accordance to this, silencing of NOX4 diminished the expression of several stem cell markers in U3031MG: CD133/PROM1, OLIG2, NESTIN and SOX2 (Fig. 5E). Even though, the GCS tested did not increase the stem cell frequency in response to TGFβ, the number of spheres formed by U3031MG and U3017MG cells increased after TGFβ treatment, and this increase was impaired after silencing of NOX4 (Fig. 5C,D). In the patient‐derived U3031MG GSCs that were mainly used in this study, 90% of the cell population was CD44+ (Fig. S5e); after TGFβ treatment, we could observe a mild increase in the CD44 expression, measured as surface protein intensity using flow cytometry, an effect that was impaired when we used the siRNA pool against NOX4 or the single siNOX4#8 (Fig. 5F). These results demonstrated a novel role of NOX4 in mediating GSC self‐renewal and maintenance of their stem cell phenotype downstream of TGFβ.

### 3.5 TGFβ signalling modulates GSC metabolism in a NOX4‐dependent manner through the NRF2 pathway

**Fig. 6:**
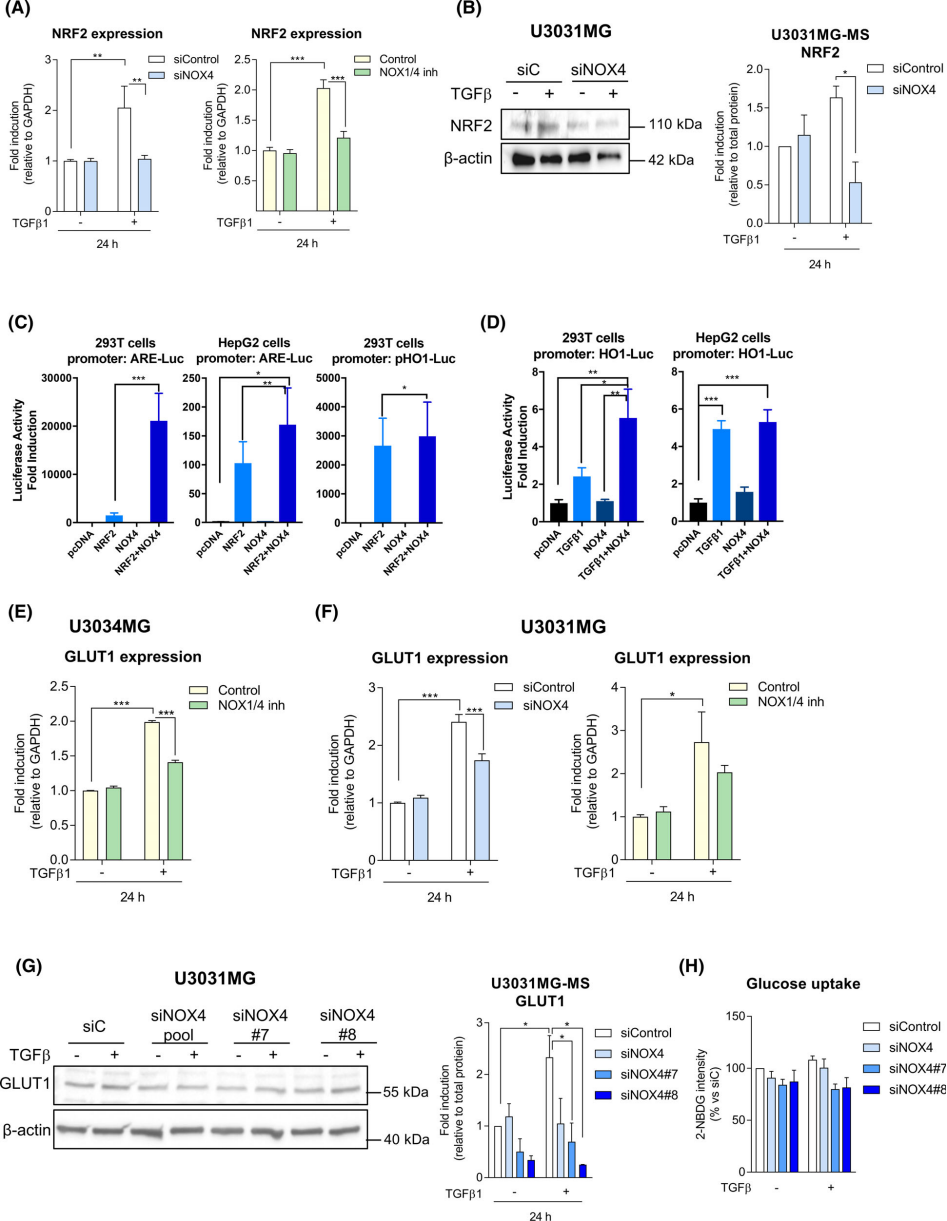
TGF‐β regulates NRF2 in an NOX4‐dependent manner. (A) NRF2 mRNA expression levels analysed by qPCR: U3031MG cells were transiently transfected with control (siControl) or NOX4 (siNOX4, pool of four siRNA sequences) siRNAs and stimulated with TGFβ1 for 24 h (left), or NOX4 function was inhibited by NOX1/4 inhibitor (right); data represent the mean ± SEM (*n* = 2 independent experiments and each with technical triplicate); statistics: two‐way ANOVA test, Tukey’s multiple comparison. (B) U3031MG cells were transiently transfected with control (siControl) or NOX4 (siNOX4, pool of 4 siRNA sequences) siRNAs and stimulated with TGFβ1 for 24 h, immunoblot of NRF2, β‐actin is used as a loading control; a representative experiment is shown and the quantification of four experiments is plotted as mean ± SEM; statistics: two‐Way ANOVA test, Sidak’s multiple comparison (C) NRF2‐responsive ARE‐luciferase reporter assay in 293T and HepG2 cells, NRF2‐responsive HO1‐luciferase reporter assay in 293T cells, data represent the mean ± SEM (*n* = 2–3 independent experiments and each with technical quadruplicate); statistics: one‐way ANOVA test, Tukey’s multiple comparison. (D) NRF2‐responsive HO1‐luciferase reporter assay in 293T cells and HepG2 cells; cells were transiently transfected with NOX4 and stimulated with TGFβ1 or 24 h prior to the measurement of luminescence, data represent the mean ± SEM (*n* = 2 independent experiments and each with technical quadruplicate); statistics: one‐way ANOVA test, Tukey’s multiple comparison. (E) GLUT1 mRNA expression levels analysed by qPCR: U3034MG cells were stimulated with TGFβ1 for 24 h in the presence or absence of the NOX1/4 inhibitor, data represent the mean ± SEM (*n* = 2 independent experiments, each with technical triplicate); statistics: one‐way ANOVA test, Tukey’s multiple comparison. (F) GLUT1 mRNA expression levels analysed by qPCR: U3031MG cells were transiently transfected with control (siControl) or NOX4 (siNOX4, pool of four siRNA sequences) siRNAs and stimulated with TGFβ1 for 24 h (left); U3031MG cells were stimulated with TGFβ1 for 24 h in the presence or absence of the NOX1/4 inhibitor (right); data represent the mean ± SEM (*n* = 2–3 independent experiments and each with technical triplicate), statistics: one‐way ANOVA test, Tukey’s multiple comparison. (G) U3031MG cells were transiently transfected with control (siControl), NOX4 (siNOX4, pool of four siRNA sequences) siRNAs or using two different siRNA sequences against NOX4 (siN4#7, siN4#8) and stimulated with TGFβ1 for 24 h; immunoblot of GLUT1, β‐actin is used as a loading control. (B, G) Quantification of immunoblot data using the densitometric analysis of each protein, normalised with total protein using Stain‐Free precast gels using image lab™ software (Bio‐Rad, Sundbyberg, Sweden); data represent mean ± SEM (*n* = 3–4 independent experiments), statistics: two‐way ANOVA test, Sidak’s multiple comparison. (H) Glucose uptake in U3031MG cells were transiently transfected with control (siControl), or NOX4 siRNAs, a pool of four sequences (siNOX4) or using two different siRNA sequences against NOX4 (siN4#7, siN4#8) and stimulated with TGFβ1 for 24 h. Data represent the mean ± SEM (*n* = 6 independent experiments for siNOX4, *n* = 2 for individual siRNA sequences); statistics: one‐way ANOVA test, Dunnett’s multiple comparison. (A–H) Statistical comparison indicates *P < 0.05, **P < 0.01, ***P < 0.001.

After observing that NOX4 has a role in self‐renewal and proliferation of GSCs, we further investigated if the nuclear factor E2‐related factor 2 (NRF2) could lie downstream of these NOX4‐mediated events, via a potential NOX4‐NRF2 axis. NRF2 is a transcription factor known to be regulated by both TGFβ and NOX4 in other cell types, with the capability to reprogramme the cellular metabolism in order to support the antioxidant response [54, 55]. TGFβ was able to upregulate the expression of *NRF2*; however, when NOX4 was silenced or inhibited the induced expression of NRF2 by TGFβ was abrogated (Fig. 6A, Fig. S3d), showing a key role of NOX4 in the induction of the NRF2 by TGFβ. The changes at the protein level are in accordance with the changes observed at the mRNA levels (Fig. 6B, Fig. S5a–d). Since NRF2 is a transcription factor, we wanted to analyse whether NOX4 by promoting NRF2 expression could also stimulate NRF2 transcriptional activity. In order to assess this, we used two different luciferase assays: ARE‐promoter containing several binding sites of the antioxidant response element (ARE) and the haemoxygenase 1 (HO‐1) promoter, a known NRF2 direct target. Both promoters were induced when NRF2 was overexpressed in 293T and HepG2 cells, and their activity was further enhanced when NOX4 was co‐expressed with NRF2 (Fig. 6C). Furthermore, TGFβ‐induced HO‐1 promoter activity, and this effect was increased when NOX4 was overexpressed in 293T and HepG2 cells (Fig. 6D). NRF2 is known to be a master regulator of cell metabolism, which can contribute to a switch from oxidative phosphorylation (OXPHOS) to glycolysis [56], TGFβ can induce the expression of one of the glucose transporters, GLUT1, in GBM [57]. We hypothesised that this might be NOX4‐dependent. TGFβ was able to increase the expression of GLUT1 in a NOX4‐dependent manner both at the mRNA and protein levels, as silencing or inhibiting NOX4 reduced TGFβ‐induced GLUT1 expression (Fig. 6E–G, Figs S3d and S5c,d). We also analysed *GLUT3* expression, which was not affected by TGFβ, but its basal levels were reduced (Fig. S3d); it is worth to note that its expression was very low, which might be because the GCS used are from mesenchymal origin, and GLUT3 has been shown to be addictive in GBMs of classical and proneural subtype [58]. Even though we observed that TGFβ induced GLUT1 expression, there was no significant increase in the glucose uptake after TGFβ treatment; interestingly, we observed that silencing of NOX4 resulted in a slight decrease of glucose uptake in GSCs (Fig. 6H). These results show that NOX4 downstream of TGFβ regulates the expression of NRF2 and GLUT1; moreover, NOX4 *per se* enhances NRF2 transcriptional activity.

### 3.6 The use of an antioxidant partially mimics the effect of NOX4 inhibition

We wondered whether TGFβ effects in proliferation, stemness and gene expression could be blocked not only by specific inhibition or silencing of NOX4, but also by using a more general antioxidant, such as butyl‐hydroxyanisole (BHA) or *N*‐acetyl cysteine (NAC), which would compensate the ROS production. We observed that TGFβ‐induced proliferation could be impaired when cells were co‐treated with BHA, and the increase in number of spheres after TGFβ treatment was also affected by the use of antioxidants (Fig. S6a,b). However, TGFβ‐induced expression of *NOX4*, *LIF*, *NESTIN*, and *GLUT1* could not be reversed by BHA or NAC (Fig. S6c,d). The use of antioxidants mimics the functional effects of NOX4 inhibition; however, the antioxidants did not impair the gene expression changes induced by TGFβ for the studied markers. This may be explained because the antioxidants have a broader effect on the cell, act in different compartments and affect more signalling pathways compared with the more targeted inhibition of NOX4.

### 3.7 NOX4 overexpression recapitulated TGFβ effects in glioblastoma stem cells

**Fig. 7:**
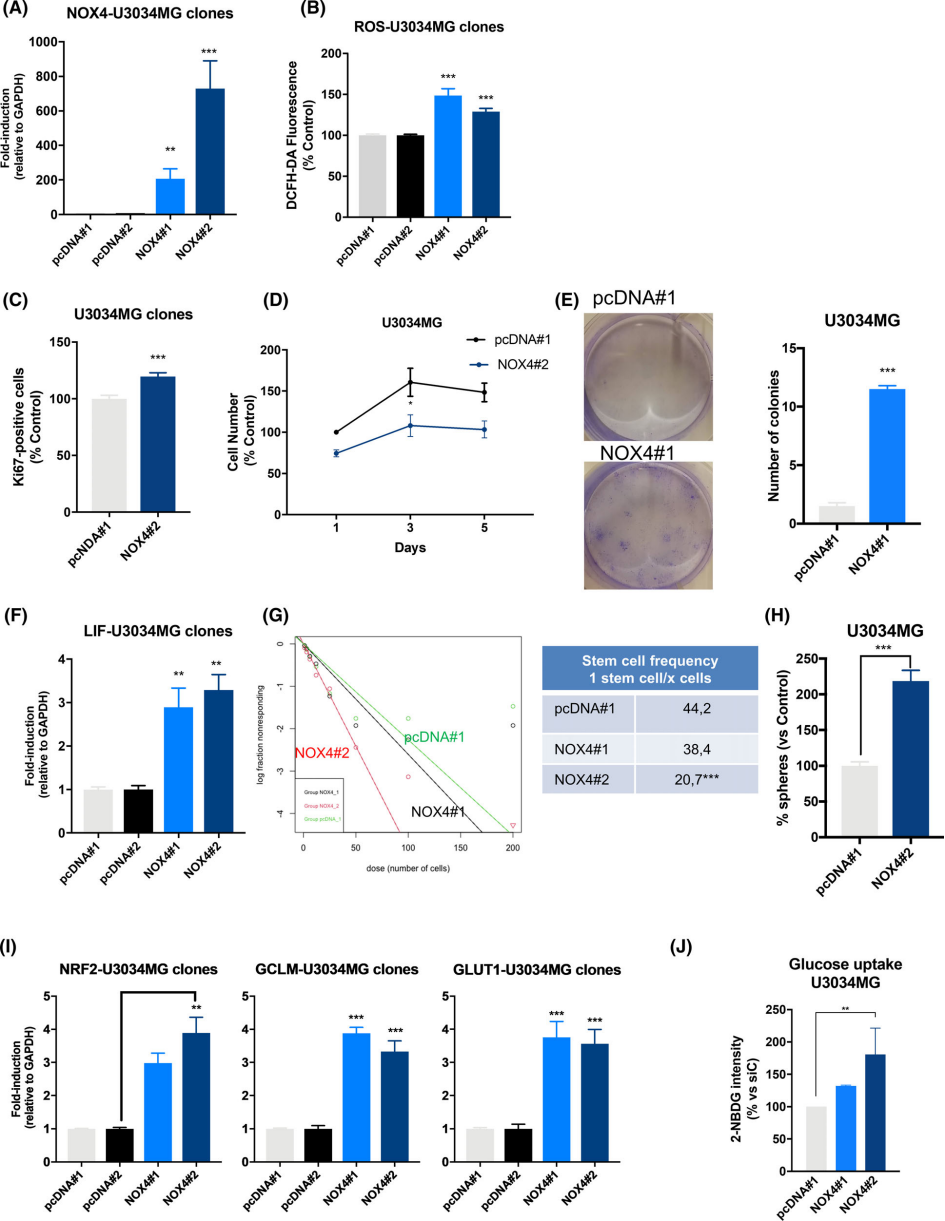
NOX4 overexpression mimics TGFβ effects in GSCs. U3034MG cells stably overexpressing the empty vector pcDNA or pcDNA‐V5‐NOX4, pool#1 and pool#2 were analysed. (A, F, I) mRNA expression levels analysed by qPCR of the indicated genes, data represent the mean ± SEM (*n* = 3 independent experiments and each with technical triplicate), statistics: one‐way ANOVA test, Dunnett’s multiple comparison. (B) Basal ROS production in the different indicated clones after 48 h of being seeded. Data represent the mean ± SEM (*n* = 3 independent experiments and each with biological triplicate), statistics: one‐way ANOVA test, Tukey’s multiple comparison. (C) Quantification of Ki67‐positive cells with respect to the control. Data represent the mean ± SEM (*n* = 3 independent experiment, 10 images per condition and experiments were quantified); statistics: two‐way ANOVA test, Bonferroni’s multiple comparison. (D) Cell viability was assayed by MTS at the indicated times, data represent the mean ± SEM (*n* = 3 independent experiment with four biological replicates); statistics: two‐way ANOVA test, Sidak’s multiple comparison. (E) Colony formation during 21 days, left panel shows representative images, right panel shows quantification. Data represent the mean ± SEM (*n* = 2 independent experiments with three biological replicates); statistics: unpaired *t*‐test. (G) Limiting dilution neurosphere assay was performed in U3034MG clones for 6 days, analysed by ELDA, the tables show the stem cell frequency and the statistical differences between both groups (*n* = 5 with 6–12 replicates each); statistics: Chi square. (H) Number of spheres was counted 6 days after seeding, when the number of spheres were counted, data represent mean ± SEM (*n* = 4 with four biological replicates); statistics: unpaired *t*‐test. (J) Glucose uptake was measured by flow cytometry; data represent mean ± SEM (*n* = 3 independent experiments); statistics: one‐way ANOVA test, Tukey’s multiple comparison. (A–J) Statistical comparison indicates *P < 0.05, **P < 0.01, ***P < 0.001.

Finally, we wanted to study whether NOX4 alone could reproduce the effects induced by TGFβ, for this reason we induced stable overexpression of NOX4 in U3034MG, one of the patient‐derived GSCs with low endogenous NOX4 expression (Fig. S3). NOX4 overexpression resulted in increased intracellular ROS levels (Fig. 7A,B) and enhanced cell proliferation (Fig. 7C) after 24 h of seeding the cells when measured by ki67 staining; however, we could not observe that NOX4 overexpressing cells had higher proliferation when measured by MTS. Interestingly, overexpression of NOX4 favoured colony formation (Fig. 7E), increasing GSCs clonogenicity. NOX4 overexpression also enhanced LIF expression and increased stem cell frequency and enhanced the number of spheres formed by GSCs (Fig. 7F–H). Interestingly, overexpression of NOX4 resulted in an increased expression of the transcription factor NRF2, concomitant with an increased expression of GLUT1 and glutamate–cysteine ligase modifier subunit, GCLM, both NRF2 targets [59] (Fig. 7I), and enhanced glucose uptake (Fig. 7J). NOX4 expression mimics TGFβ‐induced effects in GSCs. These results show for the first time the key role of this ROS‐producing enzyme in regulating stem cell capacity and the antioxidant response in GSCs.

## 4 Discussion

In glioblastoma, one of the most aggressive cancer types, current therapies are not effective and they provide a modest increase in the life expectancy of the patients [3]. In this regard, GSCs have emerged as a key element to treat GBM and considered as therapeutic targets [60]. In order to improve the current therapies against gliomas, it is vital to study and to understand the molecular mechanisms that regulate and govern the pathophysiology of GSCs. Several studies identified different signalling pathways involved in glioma, one of them being the TGFβ pathway [17]. In GBM, TGFβ is an oncogenic factor, being able to induce proliferation in GSCs, indirectly by inducing the action of PDGFB, to determine self‐renewal, by further upregulating a second cytokine, LIF, and by also promoting tumour cell migration, among other functions [18, 23, 41].

In this work, we observed that TGFβ upregulated NOX4 expression in patient‐derived GSC (Figs 1 and 2), a regulatory event that takes place in other non‐tumoural and in cancer cell types such as hepatocellular carcinoma [42, 43], with the immediate consequence of increasing the intracellular ROS levels (Fig. 2). NOX4 expression levels are increased in GBM compared with lower grade gliomas; their expression is higher in glioblastoma stem cells and transition glioblastoma cells compared with differentiated tumour cells (Fig. 1, Fig. S2). Moreover, high NOX4 expression levels also correlate with worse prognosis of GBM as previously reported [29]. NOX4 expression correlates with high levels of TGFβ ligands, a fact that we address experimentally for the first time, and its expression also correlates with different stem cell markers such as Nestin, CD44 and LIF (Fig. S1).

Neural stem cells are known to have high basal levels of ROS, helping to maintain their self‐renewal and proliferation capabilities [48]. TGFβ stimulated GSC proliferation, as previously reported by others [18]; interestingly, its effects on cell proliferation were in fact reduced when inhibiting NOX4 (Fig. 3). In this paper, we confirmed that TGFβ increased the expression of LIF in a new set of patient‐derived GSCs and show that this effect is NOX4 dependent (Fig. 4, Figs S3 and S5). In particular, GSCs *per se* had high self‐renewal characteristics with high stem cells frequency in the absence of ligand stimulation (Fig. 5). NOX4 silencing diminished stem cell frequency and it also reduced the expression of several stem cell markers such as CD133, Nestin, SOX2 and OLIG2. NOX4 silencing also impaired TGFβ capacity to increase the number of spheres formed by GSCs (Fig. 5). This suggests a key role of NOX4 and ROS to regulate self‐renewal of GSCs downstream of TGFβ as well as a role of NOX4 by itself in regulating GSC stemness.

To understand how NOX4‐ROS signalling can regulate self‐renewal and proliferation of the GSCs alone and especially downstream of TGFβ signalling, we focused our attention to the transcription factor NRF2, a master transcription factor that is able to modulate cell metabolism in order to support the antioxidant response by enhancing the pentose phosphate pathway and fatty acid oxidation, while repressing the lipid metabolic pathway [61]. We were able to elucidate that TGFβ‐induced NRF2 expression is NOX4 dependent (Fig. 6), and that NOX4 promotes NRF2 transcriptional activity. TGFβ could be enhancing GSC proliferation and stemness by modulating the antioxidant and metabolic response of GSCs in part by regulating NRF2 expression via NOX4 (Fig. 6). Finally, NOX4 regulates GLUT1 expression downstream of TGFβ, even though TGFβ does not significantly increase GSC’s glucose uptake, and the slight increase is impaired by NOX4 silencing (Fig. 6). The effects observed of TGFβ in glucose uptake disagree with previously published results in which it had been described that TGFβ induces GLUT1 expression, glucose uptake and glycolysis [57]; these discrepancies could be explained by the type of GBM cells used in the study, as GSCs cells rely mainly on oxidative phosphorylation while differentiated glioma cells have a glycolytic phenotype [62]. In our study, we used GSCs cultured in culture media that retains stem cell characteristics, while in the previous study cells were cultured in media with serum, rendering those cells more differentiated. This might explain why our GSCs cultured in stem cell media are less dependent on increasing glucose uptake even though the cells increase GLUT1 expression.

Bypassing TGFβ, overexpression of NOX4 itself is able to recapitulate the effects induced by TGFβ, such as enhanced proliferation, self‐renewal and higher levels of LIF and NRF2 which could lead to a metabolic reprogramming of the GSCs, such as increased glucose uptake, favouring their self‐renewal capacity and proliferation (Fig. 7).

In conclusion, the data presented in this work demonstrated that TGFβ pathway is able to regulate and induce NOX4 expression and, as a consequence, the ROS levels produced by this membrane oxidase are increased. Moreover, the NOX4‐produced ROS are important for several cellular functions such as proliferation, self‐renewal and glucose metabolism. Our data revealed that the TGFβ pathway regulates these functions via NOX4‐derived ROS, as well as demonstrating that NOX4 alone is a key regulator of stemness in glioblastoma.

## 5 Conclusions

In this study, we identify NOX4 as a key player downstream of TGFβ in glioblastoma cells. However, NOX4‐derived ROS regulate proliferation and stemness not only downstream of TGFβ but also act beyond TGFβ. Moreover, NOX4 protein is expressed in higher levels in glioblastoma stem cells and transition cells compared with glioblastoma differentiated cells.

## Conflict of interest

The authors declare no conflict of interest.

## Author contributions

LC conceived the project. LC, PG‐G, and IG designed the experiments. LC, PG‐G, MSD, IG, CB, KT, AM and JC‐P acquired the data. LC, PG‐G, IG, and AM analysed the data. LC, PG‐G and IG interpreted the data. LC, PG‐G, and IG drafted the article. All authors critically revised the article for important intellectual content and provided final approval prior to submission for publication.

### Peer review

The peer review history for this article is available at https://publons.com/publon/10.1002/1878‐0261.13200.

## Supporting information


**Fig. S1.** High NOX4 expression correlates with worse prognosis.
**Fig. S2.** NOX4 is expressed mainly in GSCs.
**Fig. S3.** NOX expression levels.
**Fig. S4.** NOX4 regulates TGFβ1‐induced expression of proteins related to stemness and metabolism.
**Fig. S5.** NOX4 silencing does not induce apoptosis.
**Fig. S6.** The use of antioxidants partially mimics NOX1/4 inhibitor.

**Table S1.** Lists of differentially expressed genes after 24 h of TGF‐β treatment in U3031MG and U3034MG GSCs.

## Data Availability

Transcriptomic analysis was performed by an HTA2 Affymetrix Platform, the data of this study are openly available in the ArrayExpress database at EMBL‐EBI (www.ebi.ac.uk/arrayexpress) under accession number E‐MTAB‐9076.
