# Supplementary material for: NOX4 regulates TGFβ‐induced proliferation and self‐renewal in glioblastoma stem cells
Source: Mol Oncol. 2022 Mar 14;16(9):1891–912. doi: 10.1002/1878-0261.13200 (PMC9067149; doi:10.1002/1878-0261.13200)
Supplement: Supplementary file 1 — Fig. S1. High NOX4 expression correlates with worse prognosis. Fig. S2. NOX4 is expressed mainly in GSCs. Fig. S3. NOX expression levels. Fig. S4. NOX4 regulates TGFβ1‐induced expression of proteins related to stemness and metabolism. Fig. S5. NOX4 silencing does not induce apoptosis. Fig. S6. The use of antioxidants partially mimics NOX1/4 inhibitor. [file MOL2-16-1891-s002.pdf]

Supplementary Figure 1

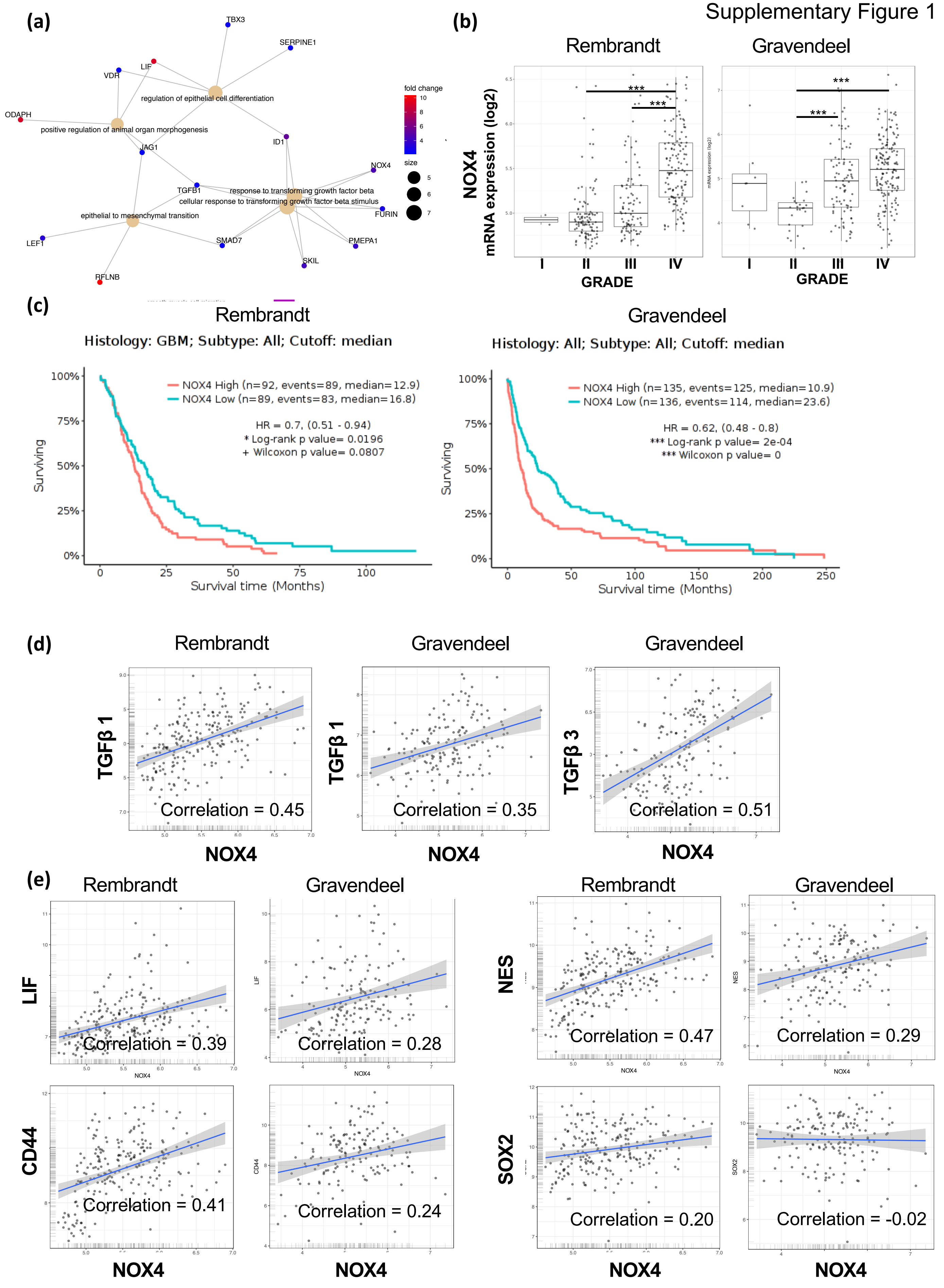

Supplementary Figure 1. High NOX4 expression correlates with worse prognosis. (a) Gene-concept network depicts the linkages between the common upregulated genes by TGFβ1 in U3031MG cells and U3034MG cells and the biological concepts as network; the size of the GO term stands for the number of upregulated genes in the TGFβ1 treated cells that are annotated based on the term. Color scale of the gene name stands for the mean fold-change in gene expression of the TGFβ1 treated cells compared to the untreated cells. (b) NOX4 mRNA expression in glioma classified according to WHO grades using different databases (REMBRANDT, left; Gravendeel, right), t-test with Bonferroni correction is shown in the pairwaise comparisons between the indicated groups, Significant differences at ***$p<0.001$. (c) Kaplan–Meier survival according to NOX4 expression, (REMBRANDT, left; Gravendeel, right). (d-e) Scatter plots of *NOX4* expression to the expression of the indicated genes (Pearson correlation analysis) using normalised mRNA expression data from REMBRANDT database and Gravendeel database (as indicated).

Supplementary Figure 2

(a)

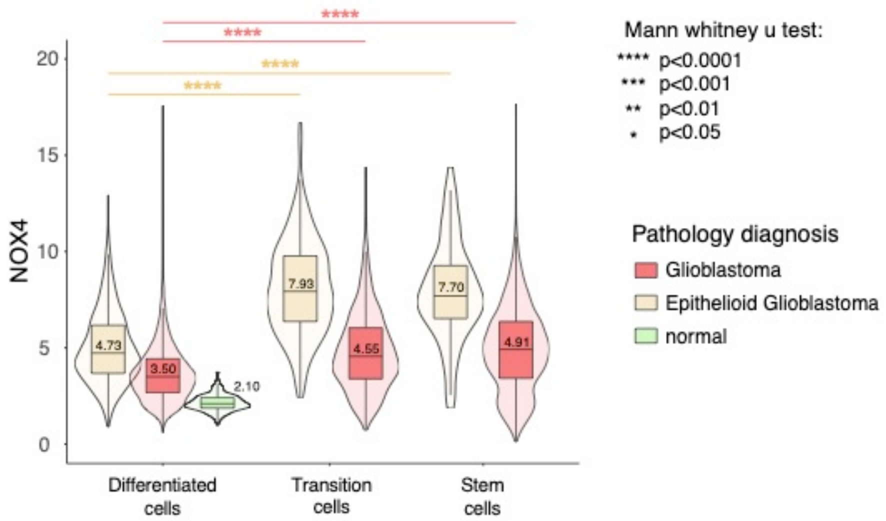

(b)

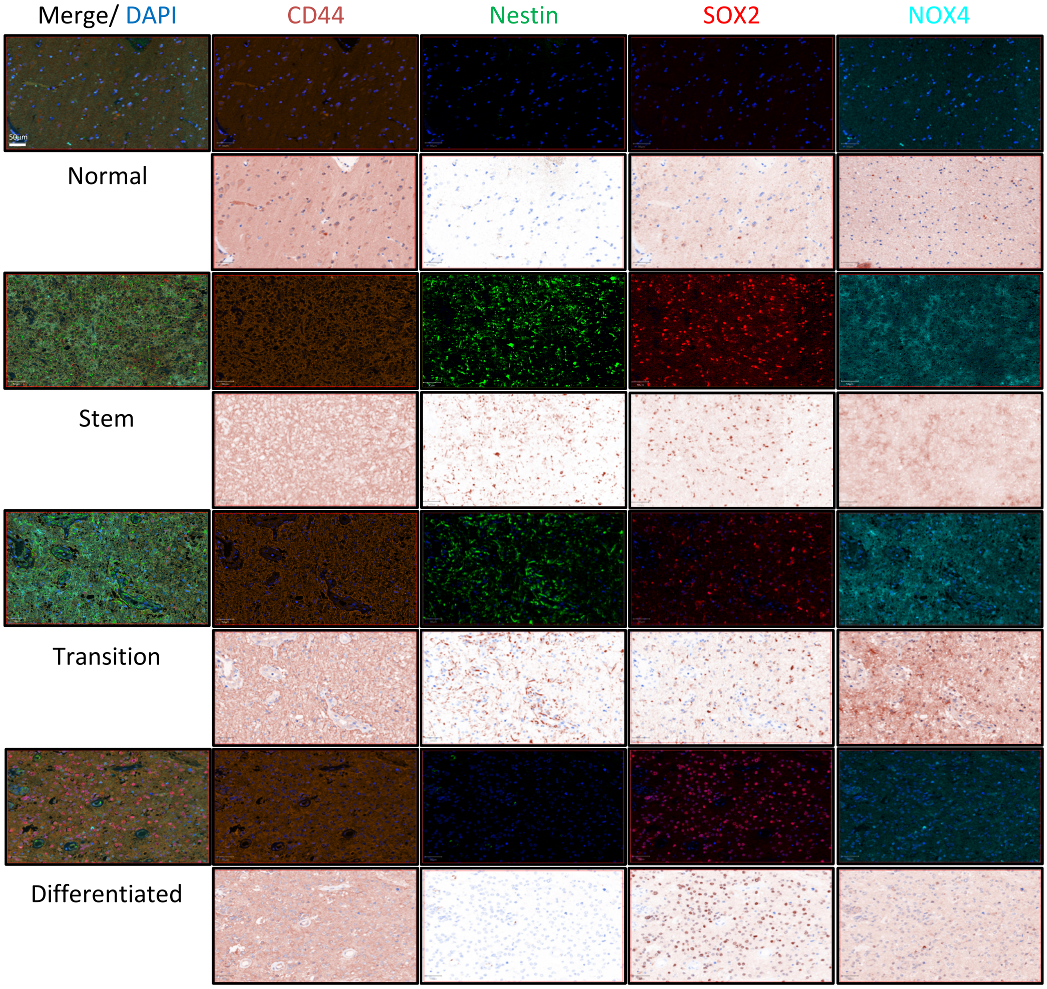

Supplementary Figure 2. NOX4 is expressed mainly in GSCs. (a) NOX4 expression per cell in normal, glioblastoma and epitheloid glioblastoma tissue samples from a tissue microarray (color-coded), plotted as a function of the three marker proteins expression in the same cell. Cells were classified to three groups, GBM_diff ($CD44^{low}$/$Nestin^{low}$/$SOX2^{low}$), GBM_transition ($CD44^{medium}$/$Nestin^{medium}$/$SOX2^{medium}$), and GBM_Stem ($CD44^{high}$/$Nestin^{high}$/$SOX2^{high}$. Significant differences at ****p<0.0001. (b) Representative images displaying staining of the four proteins, NOX4/Nestin/SOX2/CD44, in normal brain and glioblastoma tissue samples, scale bar indicates 50 µm. Significant differences at ****p<0.0001

Supplementary Figure 3

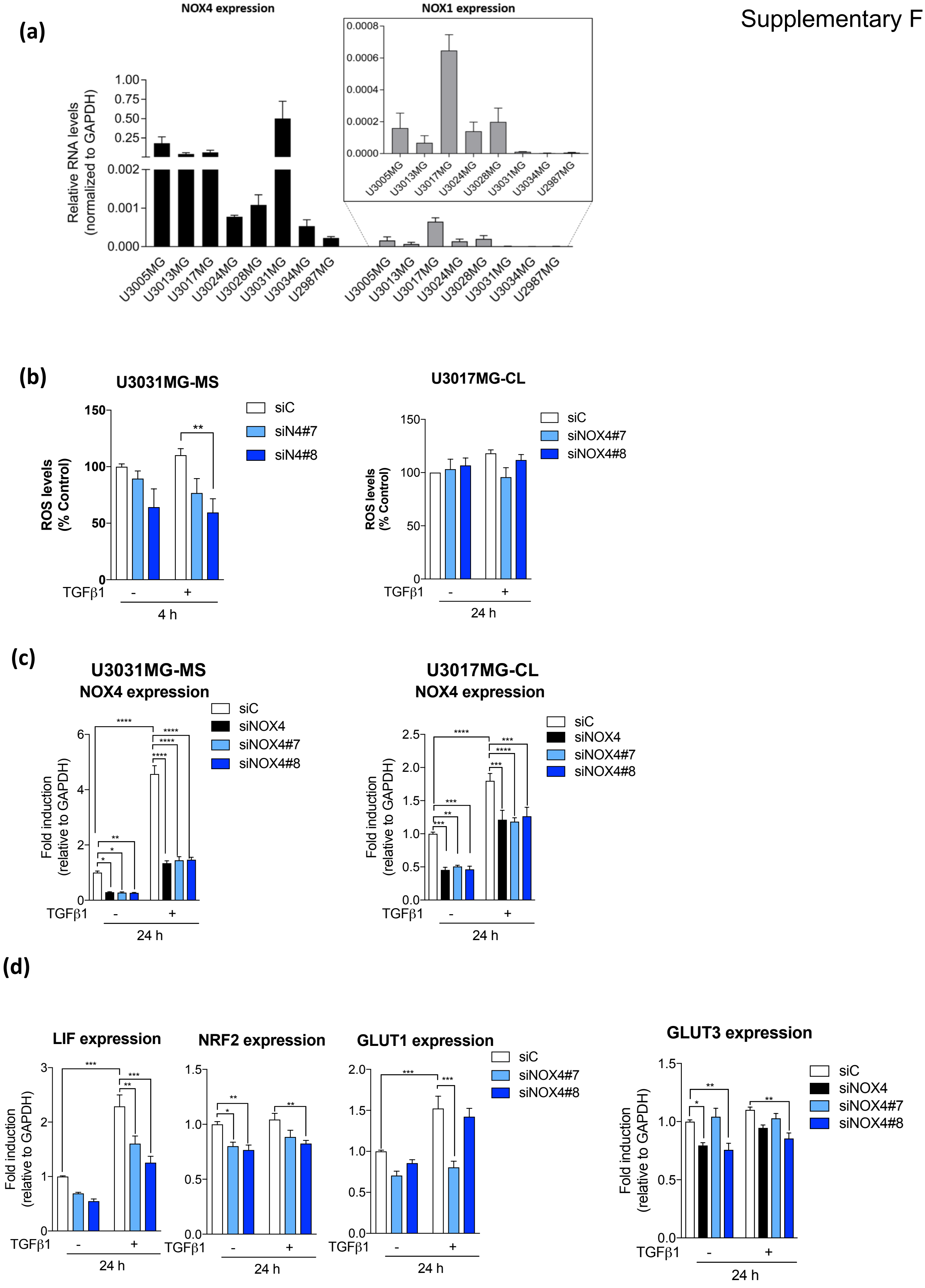

Supplementary Figure 3. NOX expression levels. (a) Expression panel of *NOX4* and *NOX1* in different patient-derived glioblastoma stem cells lines at the RNA level, normalised to *GAPDH* levels. (b) ROS production upon TGFβ1 stimulation in control (siControl) and NOX4-silenced using two different siRNA sequences against NOX4 (siN4#7, siN4#8) in U3031MG and U3017MG cells. (c) Expression of NOX4 in U3031MG and U3017MG cells transiently transfected with control (siControl), or using two different siRNA sequences against NOX4 (siN4#7, siN4#8), or a pool of 4 siRNA against NOX4; and stimulated with TGFβ1 for 24h. (d) Expression of the indicated genes in U3031MG cells transiently transfected with control (siControl), or using two different siRNA sequences against NOX4 (siN4#7, siN4#8), or a pool of 4 siRNA against NOX4; and stimulated with TGFβ1 for 24h. Data represents the mean ± s.e.m. (n=3 independent experiments and each with biological triplicate). Statistical comparison indicates ** $p < 0.01$, *** $p < 0.001$.

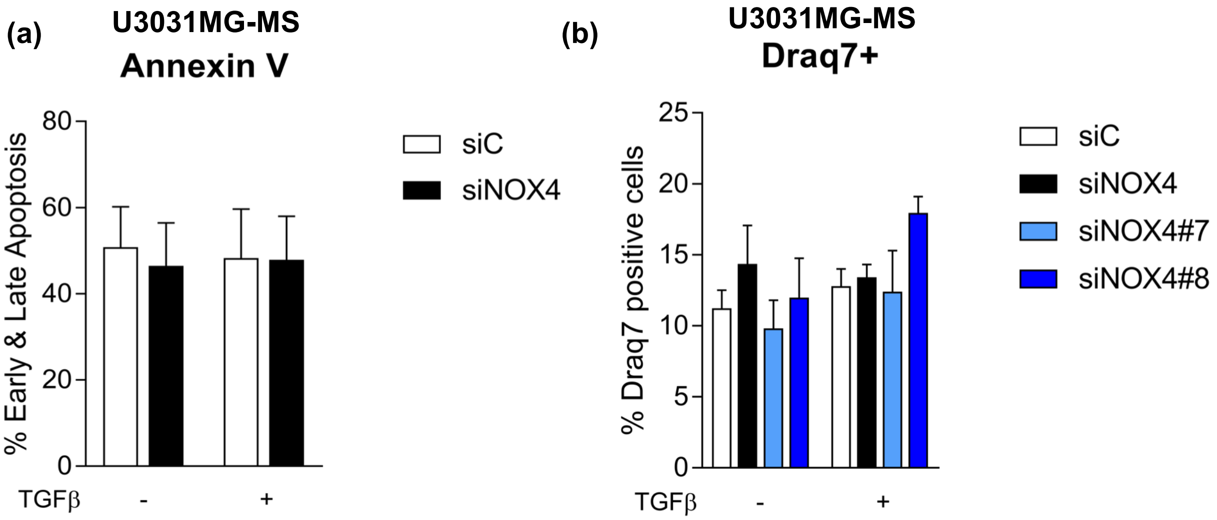

Supplementary Figure 4. NOX4 silencing does not induce apoptosis. (a-c) U3031MG cells were transiently transfected with control (siControl) or NOX4 (siNOX4) siRNAs and stimulated with TGFβ1 for 24h. (a) Apoptosis was measured by AnnexinV/Draq7 staining, graph shows the sum of early apoptotic cells (Annexin V+/Draq7- cells) and late apoptotic cells (Annexin V+/Draq7+ cells). Data represents mean ± s.e.m. (n=5 independent experiment). (b) U3031MG cells were transiently transfected with control (siControl), pool of 4 siRNA against NOX4 (siNOX4) siRNAs, or using two different siRNA sequences against NOX4 (siN4#7, siN4#8) and stimulated with TGFβ1 for 24h, late apoptosis is shown by Draq7+ cells). Data represents mean ± s.e.m. (n=4 independent experiment).

Supplementary Figure 5

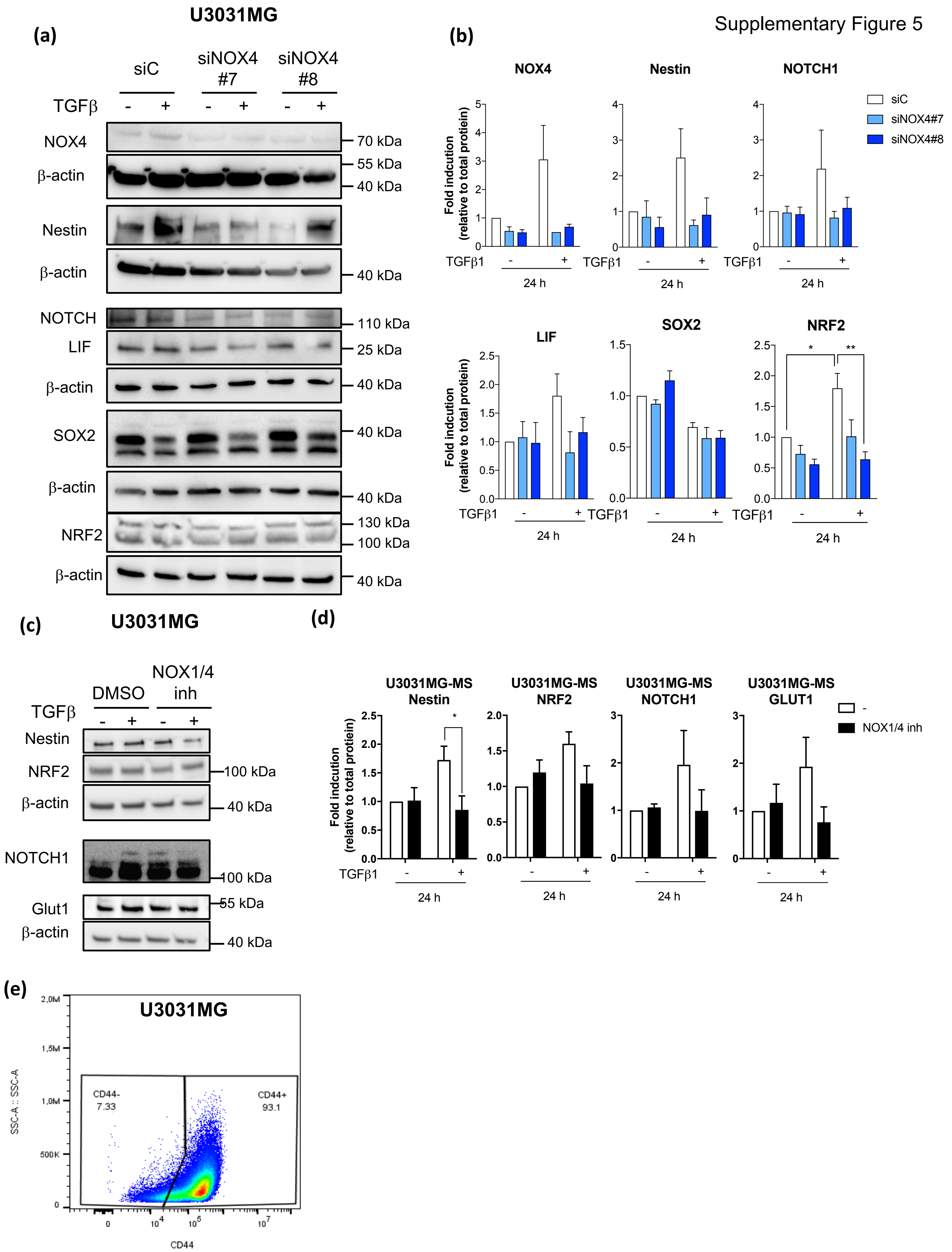

Supplementary Figure 5. NOX4 regulates TGFβ1-induced expression of proteins related to stemness and metabolism. (a) U3031MG cells transiently transfected with control (siControl), or using two different siRNA sequences against NOX4 (siN4#7, siN4#8), or a pool of 4 siRNA against NOX4; and stimulated with TGFβ1 for 24h, immunoblot of the indicated proteins, β-actin is used as a loading control, a representative experiment is shown (b) quantification of immunoblot of 3-6 independent experiments. (c) U3031MG or U3034MG cells were stimulated with TGFβ1 for 24h in with or without NOX1/4 inhibitor; immunoblot of the indicated proteins, β-actin is used as a loading control. A representative experiment is shown, (d) quantification of immunoblot of 3-6 independent experiments. (e) Flow cytometry analysis of CD44 surface marker expression in U3031MG. (b, d) Quantification of immunoblot data using the denitometric analysis of each protein, normalized with total protein using Stain Free precast gels using Image Lab™ software (BioRad, Sundbyberg, Sweden); data represents mean ± s.e.m. (n=3-6 independent experiments).

Supplementary Figure 6

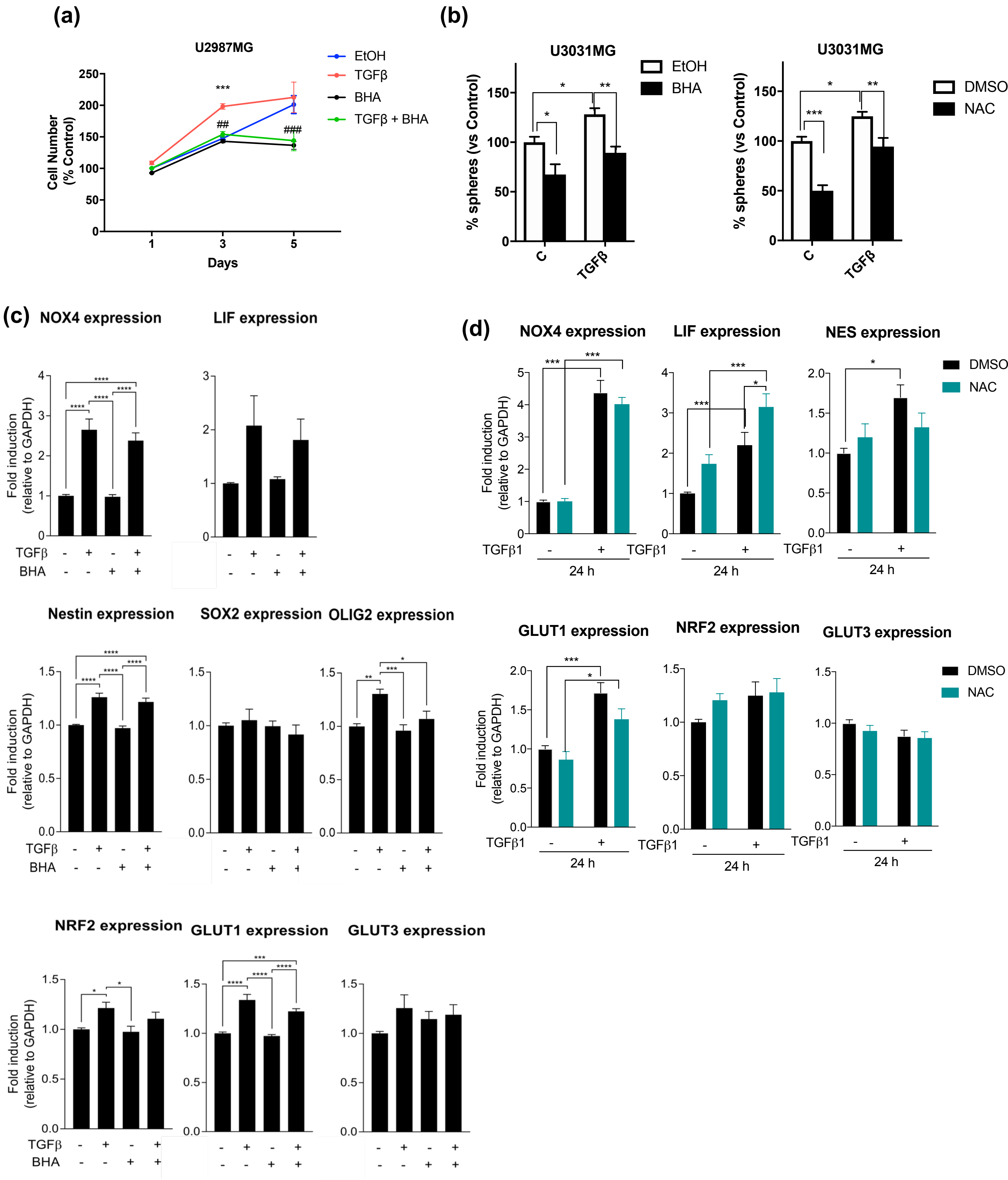

Supplementary Figure 6. The use of antioxidants partially mimics NOX1/4 inhibitor. (a) Cell proliferation was assessed by MTS in U2987MG cells treated with TGFβ1 (5 ng/ml) and BHA (200 μM) for the indicated period of times, data represents mean ± s.e.m. (n=3 independent experiment, each with 4 biological replicates). (b) Number of spheres were counted in U3031MG cells treated with TGFβ1 (5 ng/ml) and BHA (200 μM) or NAC (5 mM) for 6 days, data represents mean ± s.e.m. (n=3 independent experiment, each with 4 biological replicates). (c) mRNA expression levels analyzed by qPCR of the indicated genes in U3031MG cells treated with TGFβ1 (5 ng/ml) and BHA (200 μM) or NAC (5 mM) for 24 hours, data represents mean ± s.e.m. (n=3-4 independent experiment). Statistical comparison indicates *p<0.05, *** p < 0.001 vs Control; ## p< 0.01, ### p < 0.001 calculated vs Control-TGFβ.
